# Extensive horizontal gene transfers between plant pathogenic fungi

**DOI:** 10.1186/s12915-016-0264-3

**Published:** 2016-05-23

**Authors:** Huan Qiu, Guohong Cai, Jing Luo, Debashish Bhattacharya, Ning Zhang

**Affiliations:** Department of Ecology, Evolution, and Natural Resources, Rutgers University, Foran Hall, 59 Dudley Road, New Brunswick, New Jersey 08901 USA; National Animal Disease Center, USDA, 1920 Dayton Ave, PO Box 70, Ames, Iowa 50010 USA; Department of Plant Biology and Pathology, Rutgers University, Foran Hall 201, 59 Dudley Road, New Brunswick, New Jersey 08901 USA; Department of Biochemistry and Microbiology, Rutgers University, 76 Lipman Drive, New Brunswick, New Jersey 08901 USA

**Keywords:** Horizontal gene transfer, Pathogenesis, Magnaporthales, *Colletotrichum*, Carbohydrate activating enzymes

## Abstract

**Background:**

Horizontal gene transfer (HGT) plays an important role in the adaptation of lineages to changing environments. The extent of this process in eukaryotes, however, remains controversial. The most well-known and dramatic form of HGT represents intracellular gene transfer from endosymbionts to the host nuclear genome. Such episodes of transfer typically involve hundreds of genes and are thought to be possible only in the case of endosymbiosis.

**Results:**

Using a conservative phylogenomic approach, we analyzed genomic data from the fungal pathogen *Magnaporthiopsis incrustans* in the order Magnaporthales and identified two instances of exclusive sharing of HGT-derived gene markers between Magnaporthales and another lineage of plant-pathogenic fungi in the genus *Colletotrichum*. Surprisingly, inspection of these data demonstrated that HGT is far more widespread than anticipated, with more than 90 genes (including 33 highly supported candidates) being putatively transferred between Magnaporthales and *Colletotrichum*. These gene transfers are often physically linked in the genome and show more than two-fold functional enrichment in carbohydrate activating enzymes associated with plant cell wall degradation.

**Conclusions:**

Our work provides a novel perspective on the scale of HGT between eukaryotes. These results challenge the notion that recognized HGT plays a minor role in the evolution of fungal lineages, and in the case we describe, is likely implicated in the evolution of plant pathogenesis. More generally, we suggest that the expanding database of closely related eukaryotic genomes and the application of novel analytic methods will further underline the significant impact of foreign gene acquisition across the tree of life. Major lifestyle transitions such as those accompanying the origin of extremophily or pathogenesis are expected to be ideal candidates for studying the mode and tempo of HGT.

**Electronic supplementary material:**

The online version of this article (doi:10.1186/s12915-016-0264-3) contains supplementary material, which is available to authorized users.

## Background

Horizontal gene transfer (HGT) is a major force driving the evolution of prokaryotes as well as eukaryotes [1]. Extensive gene transfer has led to the concept of a ‘web of life’ or ‘network of life’ instead of the traditional view of a bifurcating tree of living things [2–4]. In eukaryotes, HGT is best exemplified by organellogenesis, which is accompanied by the transfer of hundreds of genes from endosymbionts to the host nuclear genome [1, 5]. In spite of the prevalence of HGT in eukaryotes [1, 3], such massive gene transfers from single sources are thought to be specifically associated with endosymbiosis [1]. Other instances of HGT typically involve a smaller number of genes derived from diverse phylogenetic sources.

From the perspective of reconstructing species relationships, HGT can create gene reticulation that misleads phylogenies (e.g., [6, 7]). On the other hand, HGT represents a form of rare genomic change [8] that can be used as a phylogenetic marker [9], or more generally to understand how selection distributes valuable “genetic goods” across the tree of life. Here, we studied the extent and impact of HGT in Magnaporthales fungi, using the following simple guiding principle: generally, fungi (including Magnaporthales) contain limited amounts of foreign genes derived from distantly related sources (e.g., [10–12]), whereas gene transfer highways exist that allow massive gene exchanges between fungal lineages (e.g., [13–15]). In this context, we hypothesized that two unrelated fungal species are unlikely to acquire the same HGT gene marker from the same (or closely related) donor species via independent events. When found, the more likely explanation is that the shared marker gene was transferred via HGT between the two species. The framework for this study is a recently generated comprehensive Magnaporthales genome database generated by our group [16].

Magnaporthales is a monophyletic order in the subphylum Pezizomycotina in the Ascomycota. This order contains approximately 200 species in three major lineages that include saprobes on submerged wood as well as pathogens that infect roots and above ground tissues of monocot plants [16]. The latter include the well-studied rice blast fungus *Pyricularia oryzae* (=*Magnaporthe oryzae*) that has devastating, worldwide impacts on food production [17]. Due to incorrect morphological identification, the rice blast fungus had been placed in the genus *Magnaporthe* and was known as *Magnaporthe grisea* and *Magnaporthe oryzae*. The *Pyricularia*/*Magnaporthe* Working Group established under the auspices of the International Commission on the Taxonomy of Fungi now recommends using *Pyricularia oryzae* for this species, which is the older and correct name for this fungus. In spite of the urgent need to ameliorate the damaging impacts of Magnaporthales on crops, the origin and genetic basis of pathogenicity in this lineage remain poorly understood.

Here, we show that Magnaporthales share two HGT gene markers with *Colletotrichum*, a large genus in the order Glomerelalles that includes anthracnose pathogens of various plants [18–21]. This HGT connection inspired us to dig deeper and resulted in the discovery of massive gene transfers between these two lineages. We examine the nature and functional significance of HGTs between Magnaporthales and *Colletotrichum* fungi and find evidence for its role in enhancing plant pathogenicity.

## Results and discussion

### Overview of Magnaporthales genomes

**Fig. 1:**
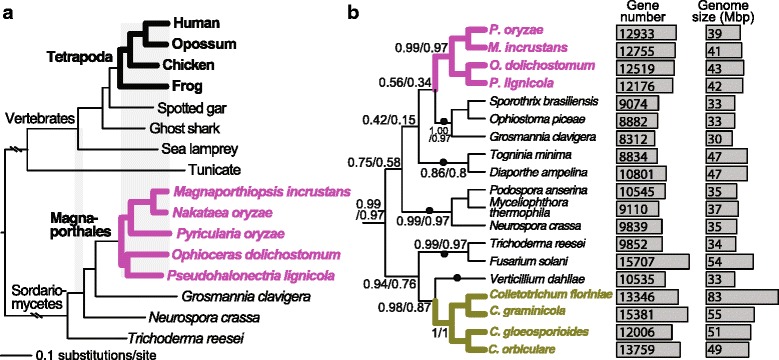
Comparative analysis of Magnaporthales genomes. **a** Evolutionary rate comparison between Sordariomycetes and vertebrates. All interior nodes have 100 % bootstrap support using a multi-protein concatenated dataset. Magnaporthales and vertebrates are highlighted using thick branches in pink and black, respectively. **b** Phylogenetic relationships among 19 lineages of Sordariomycetes, showing their genome sizes (Mbp) and predicted gene numbers. The outgroup species are not shown in this phylogeny. All interior nodes have 100 % bootstrap support using a multi-protein concatenated dataset (shown in Additional file 1). The numbers shown at the selected nodes are gene-support frequencies/internode certainty values. The black dots mark the five branches at which independent gene losses are required to explain Magnaporthales-*Colletotrichum* gene sharing under the assumption of vertical gene transmission

Magnaporthales comprises a group of fungal lineages with an evolutionary depth comparable to tetrapods (i.e., human-frog divergence; Fig. 1a). The Magnaporthales lineages possess comparable genome sizes (39–42 Mbp) and total gene numbers (12–13 K), which are typical of Sordariomycetes (Fig. 1b). To reconstruct a robust Sordariomycetes phylogeny, we identified 1453 highly conserved single-copy genes across 22 taxa (see Methods). A maximum likelihood (ML) tree built using multi-protein data comprising 20 % of the genes (291 genes and 226,915 amino acids positions) with the strongest phylogenetic signal (see Methods) resulted in a topology with 100 % bootstrap support for all interior nodes (Fig. 1b). This result is generally consistent with previous phylogenies that showed a sister group relationship between Magnaporthales and Ophiostomatales (e.g., [16, 22]).

Extended majority rule consensus and majority rule consensus (MRC) trees built using the corresponding 291 single-gene ML trees resulted in the same topology (Fig. 1b). Of the 11 internodes that define or link orders (Fig. 1b), 10 internodes have more than 50 % gene-support frequencies (GSF) or are supported by more than 50 % (146) of the single-gene ML trees (Fig. 1b). All of these internodes have more than 0.3 internode certainties (IC, see [23] for details), suggesting the defined bipartitions are more than four times more likely to exist than the most likely alternative bipartitions. The same topology and ML bootstrap support values were obtained when using the 583 (40 %) genes with the strongest phylogenetic signal and when using the full set of 1453 genes, although with decreasing GSF and IC values (Additional file 1). These results show that Magnaporthales and *Colletotrichum* are distinct lineages separated in the tree by multiple, well-defined Sordariomycetes lineages.

### HGT marker genes derived from non-Pezizomycotina sources

To search for HGT candidates, we employed a phylogenomic approach to build single-gene phylogenies for protein sequences from the specified query species. This approach is conservative because many genes do not lead to highly supported phylogenies (or no phylogenies at all) for different reasons such as lack of phylogenetic signal, short sequence length, and few detectable homologs in the database (see Methods for details). From the available Magnaporthales genomes, we used *Magnaporthiopsis incrustans* (a grass pathogen in Magnaporthales) as a representative species. We used the *M. incrustans* proteins as query against a local database that included NCBI RefSeq (version 55) and genome and transcriptome data from 110 Pezizomycotina species (Additional file 2). We identified three instances in which *M. incrustans* genes and their Magnaporthales orthologs were derived from non-Pezizomycotina (NP) sources via HGT (Additional file 3) with 85 % or more SH-like branch support [24] and 85 % or more UFboot support [25]. Limited numbers of foreign gene candidates were previously reported in its sister lineage *Pyricularia oryzae* [10, 12, 15, 26].

**Fig. 2:**
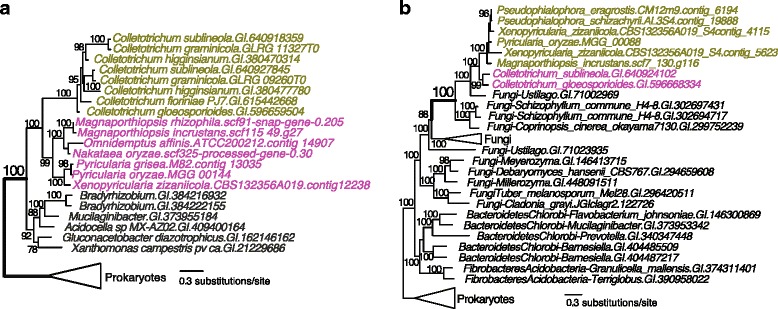
Exclusive sharing of non-Pezizomycotina-derived horizontal gene transfer gene markers in Magnaporthales and *Colletotrichum*. **a** Maximum likelihood (ML) tree of a major facilitator superfamily transporter. **b** ML tree of a putative alpha-1,2-mannosidase that participates in carbohydrate transport and metabolism

When allowing the NP-derived foreign genes to be shared with one other Pezizomycotina genus, we identified two NP-derived genes that are exclusively shared between *M. incrustans* (and Magnaporthales orthologs) and *Colletotrichum* (Fig. 2). An example is the monophyly of the Magnaporthales and *Colletotrichum* major facilitator superfamily transporter proteins that are nested within bacterial homologs (Fig. 2a and Additional file 4). The other case represents the exclusive sharing of a putative alpha-1,2-mannosidase that is derived from distantly related fungal lineages (Fig. 2b and Additional file 4). These two instances of exclusive gene sharing were confirmed using a two-way phylogenomic approach. The principle behind this method is analogous to the reciprocal-best hit approach widely used with BLAST searches. More specifically, in this case, we subjected the *Colletotrichum* sequences in Fig. 2a, b to our phylogenomic pipeline to search its sister lineages and recovered exclusive gene sharing with Magnaporthales (see Methods for details).

### Extensive gene transfer between Magnaporthales and *Colletotrichum*

Given the overall paucity of NP-derived genes in *M. incrustans* and two instances of exclusive sharing of such foreign gene markers with *Colletotrichum*, we tested the magnitude of gene transfers between *M. incrustans* and *Colletotrichum* using the two-way phylogenomic approach. Out of 9154 single gene phylogenies generated using *M. incrustans* proteins as queries, we identified 93 (1.0 %) *M. incrustans* genes with a *Colletotrichum* provenance with 85 % or above SH-like branch support [24] and 85 % or above UFboot support [25] (Additional file 5). These 93 candidates represent 89 distinct transfer events followed by independent duplications of four different genes (Additional file 5). These HGTs are located in relatively long *M. incrustans* contigs (coding ≥ 5 genes) and have orthologs in other Magnaporthales species. In 91 % (86/93) of the cases, at least one of the associated *Colletotrichum* genes is located in contigs or scaffolds encoding five or more genes. In 80 % (75/93) of the instances, shared genes are present in two or more *Colletotrichum* species. Transfers of five genomic segments comprising 2–3 HGTs were identified between the two lineages (Additional file 5). In all but one case, only limited regions of the entire length of contigs were impacted by HGT in both lineages. One example is the transfer of a two-gene Magnaporthales segment to the common ancestor of *Colletotrichum*. The phylogenies of the two genes with Magnaporthales-*Colletotrichum* groupings are shown in Additional file 6. These results, corroborated by the overall high quality of the fungal genome data, suggest that most of the identified HGT instances between Magnaporthales and *Colletotrichum* are not explained by sequence contamination.

### The nature and significance of HGT between Magnaporthales and *Colletotrichum*

**Fig. 3:**
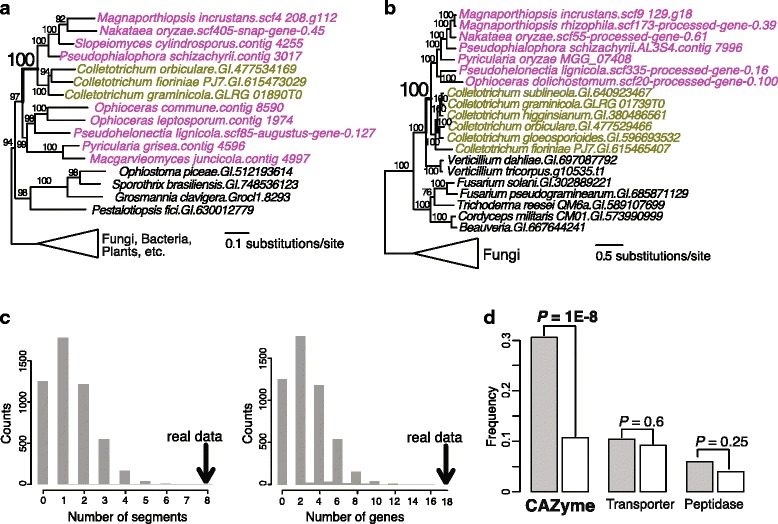
The nature of horizontal gene transfer (HGT) between Magnaporthales and *Colletotrichum*. **a** Maximum likelihood (ML) tree of a putative dimethylaniline monooxygenase. This phylogeny provides an example of a gene transfer from Magnaporthales to *Colletotrichum*. **b** ML tree of a NACHT and TPR domain-containing protein. This phylogeny provides an example of a gene transfer from *Colletotrichum* to Magnaporthales. **c** Random sampling analysis of HGT gene clustering in the *M. incrustans* genome. We randomly sampled 93 genes from the *M. incrustans* data 5000 times (see Methods) and the number of genomic segments derived from these replicates (represented by the histogram) ranged from 0 to 7. In over 99.9 % (4955) of the replicates, six or less genomic segments resulted. Therefore, the chance is less than 0.1 % to generate the eight genomic segments that were observed in the empirical data (the thick black arrow). Similarly, the range of the genes that were included in the genomic segments was 0–14 with over 99.9 % of the gene numbers being 12 or less. Therefore, the chance is less than 0.1 % to generate a total of 18 genes that are contained in genomic segments. These results suggest that the enrichment of physical linkage in our HGT data cannot be explained solely by chance. **d** The proportion of carbohydrate-activating enzymes, transporters, and peptidases among the HGT set (gray color) in comparison to those in complete-genome data (white color). The results of significance test are indicated for each comparison

Of the 93 putative instances of HGT, 45 likely resulted from gene transfers from Magnaporthales to *Colletotrichum* (Additional file 5). One example is the phylogeny of a putative dimethylaniline monooxygenase in which *Colletotrichum* sequences are nested within homologs from Magnaporthales (Fig. 3a and Additional file 4). Another 19 HGT instances were in the opposite direction (Additional file 5) including a NACHT and TPR domain-containing protein, whose phylogeny shows Magnaporthales to be nested within *Colletotrichum* and its sister-group lineage *Verticillium* (Fig. 3b and Additional file 4). The directions of gene transfers for the remaining instances are unclear.

About one-quarter of the gene transfers occurred in the stem lineage of Magnaporthales (e.g., Figs. 2a and 3b, and Additional file 4). Considering the relatively recent emergence of *Colletotrichum*, these HGTs likely occurred between the Magnaporthales common ancestor and an ancient lineage leading to extant *Colletotrichum*. Other HGT instances occurred more recently and are restricted to particular Magnaporthales lineages (e.g., Fig. 3a and Additional file 4). Given the uncertainties that result from the varying sequencing depth and differential gene loss among Magnaporthales clades, predictions about the timing of gene transfer should be treated with caution. Nevertheless, these results strongly suggest that Magnaporthales exchanged genes with the lineage leading to modern-day *Colletotrichum*.

We identified eight *M. incrustans* genomic segments (containing 18 genes) that contain two or more physically linked genes of HGT origin (allowing one intervening non-HGT gene) (Additional file 5). We manually examined the genomic locations of the relevant *Colletotrichum* genes associated with the five genomic segments without non-HGT interruption (discussed earlier). In almost all cases, the corresponding genomic segments were also found in *Colletotrichum* genomes. Random sampling 18 genes (5000 times) from the 9154 *M. incrustans* genes with single-gene phylogenies showed that the physical linkage of HGT genes is significantly more than expected by chance alone (Fig. 3c). A similar result was obtained when using the *Ophioceras dolichostomum* (instead of *M. incrustans*) proteome as the input for the two-way phylogenomic analysis (Additional file 7). A total of 51 HGTs (51 distinct transfer events) were inferred between *O. dolichostomum* and *Colletotrichum* (Additional file 8). These results suggest that HGT between Magnaporthales and *Colletotrichum* often occurred as segmental transfers involving more than one gene.

We then asked, what is the functional significance of HGT between Magnaporthales and *Colletotrichum*? From the perspective of taxonomy, out of the 1453 highly conserved single-copy orthologous genes that were identified across 22 Pezizomycotina lineages (see Methods), none were implicated in HGT. This suggests that Magnaporthales-*Colletotrichum* HGTs have a limited impact on highly conserved genes and likely does not pose significant challenges for the reconstruction of a fungal tree of life. From the perspective of functional impacts, we examined several functional categories associated with the plant pathogenic lifestyle, including carbohydrate-activating enzymes (CAZymes) [27] involved in cell wall degradation, membrane transporters, and peptidases involved in pathogenesis [28]. We found a 2.6-fold enrichment of CAZymes in the *M. incrustans* gene set derived from HGT (31.2 %; 29/93; regardless of direction and timing of HGT, Fig. 3d) when compared to the 9154-gene background data (11.7 %; 1075/9154). This enrichment was statistically significant (*P* = 1 × 10^–8^; χ^2^ test) and was not explained by post-HGT duplication of CAZyme encoding genes in Magnaporthales. The 29 transferred CAZymes represent 27 independent HGT events with only two genes having resulted from post-HGT gene duplication. Enrichment of CAZymes among genes that were transferred between Magnaporthales and *Colletotrichum* (*P* = 0.052; 19.6 % (10/51) in HGTs versus 11.0 % (999/9047) in genome background; χ^2^ test) were also observed when analyzing the *O. dolichostomum* genome data (Additional file 7). Weak or non-significant differences were however found in the distribution of transporter and peptidase genes (Fig. 3d and Additional file 7).

Given that DNA transfer and integration are largely independent of gene functions, these results suggest that HGTs with cell wall degradation functions were selectively retained (twice as likely than average) after insertion into host genomes. This function-driven selection is likely linked to the plant pathogenic lifestyles found in both lineages. The Magnaporthales-*Colletotrichum* HGT connection may therefore have been facilitated by a shared ecological niche and host. HGT occurs commonly between species that are in close proximity or have physical contact (e.g., [29–31]).

### Alternative explanations for Magnaporthales-*Colletotrichum* gene sharing

We examined three potential issues that might weaken our case for the 93 HGTs between *M. incrustans* and *Colletotrichum* (i.e., poor sampling and extensive gene loss among taxa, phylogenetic artifacts, and random chance). Regarding the first issue, when the corresponding genes were absent in all other Sordariomycetes lineages (e.g., Fig. 2a), the explanation for HGT due to poor sampling and extensive gene losses in closely related lineages would require the complete absence or loss of the impacted genes in all five Sordariomycetes lineages (Fig. 1b and Additional file 9: Figure S1) that were well-sampled in this study (Additional files 2 and 10). When assuming the existence of the node uniting Magnaporthales and *Colletotrichum* to be the Sordariomycetes common ancestor, a total of five gene losses are required to explain all Magnaporthales-*Colletotrichum* HGTs (HGT type I, see Additional file 9: Figure S1 for details). However, careful examination of the HGT gene trees derived from the *M. incrustans* genome data revealed a total of 33 independent HGT events [type II (4 genes), type III (12 genes), and type IV (17 genes)] that require more than five gene losses when vertical inheritance with gene loss is assumed (Additional file 9: Figures S2, S3 and S4). For HGT types II and III, the corresponding genes are present in additional Sordariomycetes lineages and form a sister group relationship (≥85 % UFboot support) to the Magnaporthales-*Colletotrichum* monophyletic clade (e.g., *Verticillium* in Fig. 3b). This leads to phylogenetic conflicts because Magnaporthales and *Colletotrichum* are separated by additional Sordariomycetes lineages in the species tree shown in Fig. 1b (see Additional file 9: Figures S2 and S3 for details). To explain these phylogenetic conflicts, one ancient gene duplication and 11 independent gene losses are required when assuming vertical inheritance and gene loss, whereas only one gene transfer (type II) and an additional gene loss (type III) are required when HGT is allowed (Additional file 9: Figures S2 and S3). We also identified HGT cases (type IV), in which *Colletotrichum* species are nested among Magnaporthales or vice versa (with ≥ 85 % UFboot support at the relevant nodes, Fig. 3a and Additional file 9: Figure S4). The phylogenetic conflicts raised in these HGTs require a total of one ancient gene duplication and 11 independent gene losses when assuming vertical inheritance and gene loss, whereas only one gene transfer (Type IV, scenario b) and an additional gene duplication (Type IV, scenario a) are required when HGT is allowed (see Additional file 9: Figure S4 for details). Whereas we cannot definitively exclude the possibility of vertical inheritance and gene loss as an explanation for each HGT candidate identified in this study, a total of 33 HGT cases (corresponding to HGT types II–IV, explained in Additional file 9) are highly unlikely to be explained by the vertical inheritance and gene loss scenario. The topologies and supporting values of these high confidence HGTs (available in Additional file 11) were confirmed via examination of gene trees generated from two-way phylogenomics and from the HGT validation procedure (see Methods). A total of 15 independent HGTs (types II–IV) were found in *O. dolichostomum* genome data (Additional file 11).

For the second issue, we applied a novel implementation of two-way phylogenomics and an additional round of phylogenomic analysis to search for and validate HGTs. These analyses involve different sequence sampling strategies (taxonomically dependent and independent sampling, and BLASTp hits sorted by bit-score and by sequence identity) and different tree building methods (FastTree and IQtree) (see Methods for details). The Magnaporthales-*Colletotrichum* HGTs are therefore unlikely to be primarily explained by phylogenetic artifacts. Regarding the third issue, it is possible that analysis of large genomic datasets might lead to observations of HGT that are explained solely by chance. However, random sampling of the Magnaporthales gene set (see Methods) is unlikely to generate as many physical linkages as we report in the empirical data (Fig. 3c and Additional file 7). The enrichment of physical linkages among HGT candidates (<0.1 % chance by random sampling, Fig. 3c and Additional file 7) is therefore unlikely to be accounted for solely by chance due to the large amount of genome data being analyzed. Likewise, the observed enrichment of CAZyme genes (*P* = 1 × 10^–8^ in *M. incrustans* data, Fig. 3d; and *P* = 5 × 10^–2^ in *O. dolichostomum* data, Additional file 7) in our HGT data is unlikely to be explained by random chance.

## Conclusions

Due to greater similarities in genomic properties such as gene structure and shared regulatory elements, HGT between closely related species is thought to be more frequent than between distantly related taxa. However, our understanding of recent HGT between closely related lineages is limited due to difficulties in distinguishing alternative scenarios (e.g., gene duplication and differential gene loss [32]) and the inability to resolve the topology of closely related gene sequences due to stochastic processes (low divergence, extensive ancestral polymorphisms) operating in single-gene phylogenies. Here, we show that well-resolved ancient HGTs can provide a powerful marker to identify candidate species to test for more recent gene transfer events. The resulting putative HGTs can be substantiated with structural and functional analyses.

What distinguishes the HGTs between Magnaporthales-*Colletotrichum* from other reported cases of intra-phylum HGT among fungi (e.g., [10–12]) is scale and magnitude. HGT is generally thought to be highly limited in fungal species [10] because of their robust chitin-rich cell walls and the loss of phagocytosis [12, 33]. The conservative estimation of 93 putative gene transfers between *M. incrustans* and *Colletotrichum* (including 33 highly supported cases) provides a new perspective on the extent of genetic exchange between fungal pathogens and in eukaryotes in general. The only other known fungal lineage displaying a similar or higher scale of HGT is the genus *Aspergillus* in the class Eurotiomycetes (e.g., [13–15]). The underlying mechanisms responsible for HGT between fungal species are well documented and include anastomosis, which can lead to physical connections between cells from different species (reviewed in [10]). In conclusion, our results provide novel insights into the evolution and pathogenicity in Magnaporthales and *Colletotrichum*, and suggest that many yet uncovered highways of HGT between closely related fungi remain to be discovered.

## Methods

### Construction of multi-protein phylogenies

To construct a genome database on Sordariomycetes phylogeny (Fig. 1b), we assembled a local database comprising complete proteomes from 19 Sordariales and 3 Pezizomycotina (Additional file 2). These data were subjected to an all-versus-all self-BLASTp search (*e*-value cut-off = 1 × 10^–10^). Orthologous groups across the 22 taxa were constructed using ORTHOMCL [34] under default setting with modifications (valueExponentCutoff = −10 and percentMatchCutoff = 40). Sequences were retrieved from the single-copy orthologous groups containing one sequence from each of the sampled taxa.

For each gene family, the sequence alignment was built using MUSCLE [35] under default settings with the poorly aligned regions being removed using TrimAl (−automated). We further applied T-COFFEE [36] to remove poorly aligned residuals (i.e., conservation score ≤ 5) within the well-aligned blocks. Sequences less than one-half of the alignment length and columns with more than 10 % gaps were also removed from the alignments. This procedure led to 1453 alignments with 22 sequences and with 150 or more amino acid positions that were used for downstream analyses.

We used the IC measurement to assess the extent of internode conflicting phylogenetic signal among the multi-gene data [23]. For each single-gene alignment, we generated a ML tree and 100 bootstrap trees using IQtree [37] under the best evolutionary model identified by the build-in model selection function (−m TEST). The extended majority rule consensus tree and tree certainty values (TC, see [23] for details) were computed for each single gene using RAxML (v8.2.4) [38]. We ranked the 1453 genes according to their phylogenetic signals (gauged by TC values) and used the ML trees from the top 291 genes (20 %) to build species trees with three different methods and measurements: (1) A MRC tree was built using the ‘consense’ function in the Phylip package (http://evolution.genetics.washington.edu/phylip.html). The GSFs for each internode of the MRC tree were expressed as bootstrap values (Fig. 1b). (2) The same 291 ML trees were used to compute the IC values under an extended majority rule consensus tree using RAxML (v8.2.4; Fig. 1b). (3) The corresponding alignments of the 291 genes were concatenated to build a multi-protein tree using RAxML (v7.2.8) [38] under the PROGAMMALGF model identified by ProtTest (v3.2) [39]. The bootstrap values were generated using 100 replicates (Additional file 1). We performed two additional analyses using the top 40 % (583) genes and the entire set of 1453 genes. The corresponding topologies and statistic estimations (SGF, IC, and ML bootstrap values) are shown in Additional file 1.

### Construction of the Sordariomycetes-vertebrate phylogeny

To compare the evolutionary rates between Sordariomycetes and vertebrates, we constructed a phylogeny (shown in Fig. 1a) using a concatenated multi-protein alignment. The genome data from 16 relevant species were described in Additional file 12. Orthologous gene families were constructed following the same procedure as aforementioned. Single-copy orthologous groups across the 16 species were identified allowing data missing in one vertebrate species and one Sordariomycetes species. A total of 813 single-gene alignments were built following the same procedure as previously described. The concatenated super-alignment (322,392 amino acids) was used from tree building using RAxML (v7.2.8) [38] under the PROGAMMALGF model. The bootstrap values were generated using 100 replicates.

### Two-way phylogenomic analysis

Protein sequences in RefSeq (version 55) were downloaded from the NCBI FTP site (ftp://ftp.ncbi.nlm.nih.gov/refseq/). When sequences were available from more than one (sub) species in a genus (e.g., *Arabidopsis thaliana* and *A. lyrata* in the genus *Arabidopsis*), the species (e.g., *A. thaliana*) with largest number of sequence were retained, whereas others (e.g., *A. lyrata*) were removed. To reduce sequence redundancy in the database, we clustered highly similar sequences (identity ≥ 85 %) among taxa from each order (e.g., primates and Brassicales), retained the longest sequence and removed all other related sequences in the same cluster using CD-HIT version 4.5.4 [40]. This step enhanced exploitation of sequence diversity from a given group by avoiding sampling from the same or closely related taxa. The Pezizomycotina sequences from the RefSeq database (version 55) were removed and replaced with more recent (RefSeq version 69) and comprehensive data listed in Additional file 2 that was downloaded from NCBI (unless otherwise mentioned). For four species (*Diaporthe longicolla*, *Diaporthe ampelina*, *Valsa mali*, and *Verticillium tricorpus*), the whole-genome assemblies downloaded from NCBI were used for protein prediction using Augustus [41] under the Magnaporthales model. Highly similar sequences (identity ≥ 85 %) among each species were removed using CD-HIT version 4.5.4 [40].

Whole-proteome data from *Magnaporthiopsis incrustans* [10] was used as query to search the aforementioned local database using BLASTp (*e*-value cut-off = 1 × 10^–5^). The top 1200 significant hits with query-hit similarity (≥30 %) for each query sequence were recorded with the default order sorted by bit scores. Representative sequences were selected in order allowing up to three sequences for each order and 15 sequences from each phylum. Within Pezizomycotina, we allowed up to three sequences to be sampled from each clade of Magnaporthales (i.e., Clade A, B, and C) [16]. In addition, up to 15 sequences were retrieved from Sordariomycetes (not counting Magnaporthales) with up to three sequences for each of the five orders, Ophiostomatales, Diaporthales, Sordariales, Hypocreales, and Glomerelalles (containing *Colletotrichum*). The sampling of sequence stopped when a total of 90 sequences were selected or the entire list of BLASTp hits was read through. In case of less than 72 sequences were selected in the first round of sampling, further rounds of sampling were carried out from the remaining BLASTp hits until the number of selected sequence reached 72 or all BLASTp hits were selected. Because sequence sampling was centered on the query sequences, this bias likely generated phylogenetic trees that do not accurately reflect the true evolutionary history of the corresponding genes. However, due to our focus on the immediate sister lineages to the query genes, the results regarding “deeper” evolution and the interrelationships among other clades in the trees were of lesser concern.

The selected representative sequences were retrieved from database and were aligned using MUSCLE version 3.8.31 [35] under default settings and trimmed using TrimAl version 1.2 [42] in an automated mode (-automated1). Columns with gaps (in ≥ 50 % sequences) were removed. The resulting alignments (length ≥ 80 amino acids) containing at least ten non-Magnaporthales were used to build phylogenetic trees using FasTree [43] under ‘WAG + CAT’ model. To achieve higher level of accuracy, we used four rounds of minimum-evolution SPR moves (-spr 4) and made ML nearest-neighbor interchanges more exhaustive (-mlacc 2 -slownni). The trees with supported monophyletic relationships (≥85 % SH-test) between query sequences and target species were searched using in-house tools.

To confirm the sister relationships to the Magnaporthales sequences, we performed a second round of phylogenomic analysis using the sequences from the non-Magnaporthales sister lineages as queries. Taking the Magnaporthales-*Colletotrichum* monophyly shown in Fig. 2a, for example, this case was identified using *M. incrustans* sequence (*scf115_49.g27*) as query through the aforementioned phylogenomic pipeline. In the two-way phylogenomic approach, the eight *Colletotrichum* sequences were subjected to the same phylogenomic procedure except that Magnaporthales was regarded as an order (i.e., with ≤ 3 sequences to be sampled from). The resulting trees were scanned for Magnaporthales-*Colletotrichum* monophyly supported with 85 % or above SH test. When one or more of the *Colletotrichum* sequence-derived trees supported the monophyly, this case was considered as a candidate of HGT. Given the variable quality of protein sequences and conservative nature of our phylogenomic procedure, we did not require all *Colletotrichum* sequence-derived trees to support the Magnaporthales-*Colletotrichum* monophyly.

Finally, the alignments from all HGT candidates were further used for tree building using IQtree [37] under the best evolutionary models that were selected using the build-in model selection function. Branch support was estimated using the ultrafast bootstrap (UFboot) approximation approach [25] with a 2000 maximum number of iterations (-nm 2000) and 2000 bootstrap replicates (-bb 2000). Trees with Magnaporthales-*Colletotrichum* monophyly supported with 85 % UFboot were manually searched and were subjected to a validation procedure (see below). The final HGT sets (93 HGTs derived from the analysis of *M. incrustans* proteome and 51 HGTs from the *O. dolichostomum* proteome, available in Additional file 11) were used for downstream analyses. The direction and timing of gene transfer were determined manually.

To cluster genes resulting from post-HGT duplication in Magnaporthales, we constructed, for each HGT gene tree, a gene family comprising the query sequence and its Magnaporthales and *Colletotrichum* sister lineages. Two or more gene families were merged into a bigger gene family if they possessed one or more shared members. HGT genes associated with the same gene family were considered as having resulted from a single HGT event.

### Validation of HGT candidates

To validate the HGT candidates identified in our two-way phylogenomic approach, we expanded the Sordariomycetes protein data used in the phylogenomic analyses in the following way: (1) We downloaded all Sordariomycetes sequences (>1.8 million) that are available in NCBI Protein database (Jan. 2016). The redundant sequences (≥99 % identify) among this dataset were removed using CD-HIT version 4.5.4 [40]. (2) To capture genes that might have been missed in automated gene predictions, we downloaded all 149 Sordariomycete genome assemblies available from the NCBI Genome database (Jan. 2016). After removing organelle genomes and Magnaporthales and *Colletotrichum* genomes, 123 nuclear genome assemblies were retained for further uses. We searched the Magnaporthales (*M. incrustans* and *O. dolichostomum*) HGT candidates against the Sordariomycetes genome assemblies using tBLASTn (*e*-value cut-off = 1 × 10^–5^). The translated peptides from the genome assemblies were parsed using an in-house script. These peptides mostly comprise fragments of complete proteins because of the presence of introns in fungal genomes. To mitigate this problem, we parsed the genomic regions (exon regions) bearing homology to the *M. incrustans* (or *O. dolichostomum*) queries from tBLASTn outputs. Genomic regions (corresponding to the same query sequences) that were less than 1 Kb apart were connected and merged into larger genomic regions. The resulting genomic regions and the corresponding query protein sequences were used for homology-based gene prediction using GeneWise [44]. GeneWise predicts exon-intron structure on the basis of homology between query protein and nucleotide data and returns the resulting protein sequences encoded in genomic sequences [44]. We collected all predicted proteins that had scores (≥25). Finally, the protein sequences derived from tBLASTn- and GeneWise-based analyses were pooled. The redundant sequences (≥99 % identify) were removed using CD-HIT version 4.5.4 [40]. (3) The Sordariomycetes proteins derived from the above two approaches were combined and used to replace the smaller Sordariomycetes protein dataset included in the database that was used in the aforementioned phylogenomic analysis.

To mitigate the effects of possible sequence sampling bias in our approach, an additional round of phylogenomic analyses were carried out using the *M. incrustans* and *O. dolichostomum* HGT candidate genes as queries. The analyses were performed following the same procedure as described above with the following modifications. (1) The top 80 hits (regardless of taxonomic origin) were kept for all downstream phylogenetic analyses. In other words, no limitations were placed on the number of retrieved sequences for an order or a phylum. The exceptions to this rule are Magnaporthales and *Colletotrichum* from which no more than five sequences were sampled. (2) In addition to the default sequence order (by bit-score) used in the BLASTp search output, we resorted the hits based on query-hit similarity in a descending order. Because very short query-hit alignments provide no information representative of complete genes (e.g., caused by shared domains), the sorting was restricted among hits with relatively long alignments (>120 amino acids). The ranking of short-alignment hits remained unchanged after resorting. This modified phylogenomic procedure was applied to all NP-derived *M. incrustans* genes and all Magnaporthales-*Colletotrichum* HGT candidates generated from the two-way phylogenomic approach. Two IQtree-derived ML trees were generated for each query, with one derived from bit score-based hit sorting (by default) and the second from the sequence similarity-based sorting.

We manually examined the phylogenetic tree pairs for each Magnaporthales query sequence that was generated by the modified phylogenomic approaches. The HGT status of Magnaporthales query sequences was rejected if one or both of its resulting gene trees did not support the assumed HGT scenario.

### Identification of CAZymes, transporters, and peptidases

To infer fungal CAZymes [27], fungal protein sequences were submitted to the BLAST server (http://mothra.ornl.gov/cgi-bin/cat/cat.cgi?tab=ORTHOLOGS) available as a part of CAZyme Analysis Toolkit [45]. The BLASTp searches were carried out with cutoff (*e*-value ≤ 1 × 10^–10^). Transporters were detected using BLASTp search (*e*-value ≤ 1 × 10^–10^) against the transporter classification database [46] (downloaded at Aug. 14, 2015). Peptidases were detected using MEROPS batch BLAST server [47] under the default setting.

### Physical clustering of HGT-derived genes

A segment of foreign genes was defined as comprising two or more HGT-derived genes that were physically linked. Considering the conservative nature of HGT detection and changes in genomic location following HGT, one intervening gene (not detected as HGT) was allowed to be present between the two HGTs. To test if the observed physical clustering of HGTs was significantly more than expected by chance alone, we randomly sampled the same number of genes (as the actual number of HGTs) from the gene population that was subjected to single-gene phylogeny construction. The multi-gene segments among these sampled data were identified and recorded. This random sampling-based analysis was repeated 5000 times. The resulting information (i.e., the number of genomic segments and number of genes in the segments) derived from actual data and randomly generated data were plotted as shown in Fig. 3c and Additional file 7.

## Additional files

Additional file 1: Species tree constructions with three multi-gene datasets. (PDF 220 kb) Additional file 2: Pezizomycotina RefSeq proteomic data included in the two-way phylogenomic analysis in this study. (PDF 157 kb) Additional file 3:
*Magnaporthiopsis incrustans* genes putatively derived from non-Pezizomycotina species via horizontal gene transfer. (PDF 173 kb) Additional file 4: The complete set of taxa included in the phylogenetic trees that are shown in Fig. 2 and Figs. 3a and 3b. (PDF 194 kb) Additional file 5: Annotation of horizontal gene transfers that are transferred between Magnaporthales (using *M. incrustans* as a reference) and *Colletotrichum*. (PDF 153 kb) Additional file 6: Phylogenies of two physically linked genes that were transferred as a genomic segment between Magnaporthales and *Colletotrichum*. (PDF 105 kb) Additional file 7: Horizontal gene transfer analysis of *Ophioceras dolichostomum* data. (PDF 101 kb) Additional file 8: Annotation of horizontal gene transfers that are transferred between Magnaporthales (using *O. dolichostomum* as a reference) and *Colletotrichum*. (PDF 140 kb) Additional file 9: Classification of Magnaporthales-*Colletotrichum* horizontal gene transfers (HGTs) and taxonomic gene mapping and HGT/gene loss counts. (PDF 384 kb) Additional file 10: Sordariomycete genome assemblies used for horizontal gene transfer validation. (PDF 102 kb) Additional file 11: Single-gene trees (Newick format) for the final sets of horizontal gene transfer (HGTs) that are transferred between Magnaporthales (using *M. incrustans* and *O. dolichostomum* as references) and *Colletotrichum* and those correspond to HGT types II–IV. (TXT 1064 kb) Additional file 12: Vertebrate and fungal genome data underlying Fig. 1a. (PDF 239 kb)

## Abbreviations

HGT: Horizontal gene transfer
CAZyme: Carbohydrate activating enzymes
GSF: Gene-support frequency
IC: Internode certainty
ML: Maximum likelihood
MRC: Majority rule consensus
NP: Non-Pezizomycotina
TC: Tree certainty
