# Supplementary material for: Extensive horizontal gene transfers between plant pathogenic fungi
Source: BMC Biol. 2016 May 23;14:41. doi: 10.1186/s12915-016-0264-3 (PMC4876562; doi:10.1186/s12915-016-0264-3)
Supplement: Additional file 1: — Species tree constructions with three multi-gene datasets. (PDF 220 kb) [file 12915_2016_264_MOESM1_ESM.pdf]

Additional file 1. Species tree constructions with three multi-gene datasets.

A

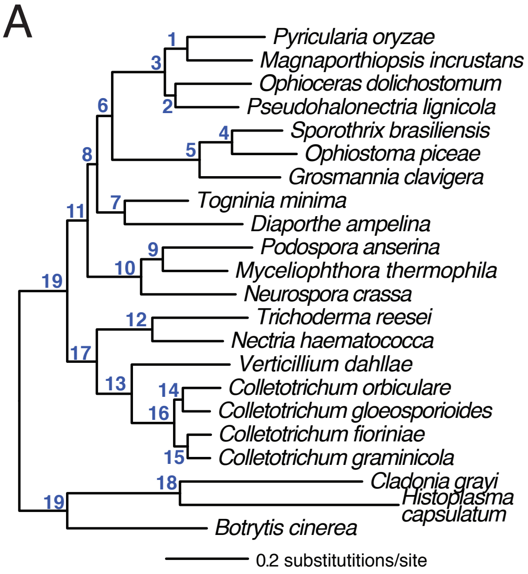

B

	291-gene dataset			582-gene dataset			1453-gene dataset		
	**Bootstrap**	**GSF**	**IC**	**Bootstrap**	**GSF**	**IC**	**Bootstrap**	**GSF**	**IC**
internode 1	100	0.48	0.07	100	0.46	0.05	100	0.42	0.07
internode 2	100	0.91	0.64	100	0.84	0.54	100	0.70	0.39
internode 3	100	0.99	0.97	100	0.99	0.98	100	0.90	0.92
internode 4	100	0.98	0.85	100	0.95	0.76	100	0.85	0.59
internode 5	100	1.00	0.97	100	1.00	0.98	100	0.98	0.97
internode 6	100	0.56	0.34	100	0.50	0.36	100	0.36	0.28
internode 7	100	0.86	0.80	100	0.78	0.75	100	0.59	0.61
internode 8	100	0.42	0.15	100	0.31	0.08	100	0.21	0.07
internode 9	100	0.79	0.40	100	0.76	0.36	100	0.65	0.24
internode 10	100	0.99	0.97	100	0.98	0.97	100	0.85	0.89
internode 11	100	0.75	0.58	100	0.64	0.47	100	0.46	0.38
internode 12	100	0.99	0.97	100	0.99	0.97	100	0.91	0.86
internode 13	100	0.98	0.87	100	0.95	0.83	100	0.83	0.72
internode 14	100	0.66	0.15	100	0.62	0.15	100	0.56	0.12
internode 15	100	0.94	0.81	100	0.92	0.72	100	0.80	0.56
internode 16	100	1.00	1.00	100	1.00	1.00	100	0.95	0.91
internode 17	100	0.99	0.76	100	0.87	0.63	100	0.69	0.53
internode 18	100	0.99	0.92	100	0.98	0.87	100	0.90	0.67
internode 19	100	0.99	0.97	100	0.98	0.95	100	0.92	0.93

(A) Maximum likelihood tree of 22 Sordariomycetes species using the 291-gene data. MRC tree and eMRC tree display the same topology. Analyses of the 585-gene data and 1453-gene data lead to the topology and similar branch lengths. (B) The internode statistics of the tree shown in panel A are based on analysis of the three multi-gene datasets. The intensity of the colors reflects the relative values across the rows. GSF: gene-support frequency. IC: internode certainty.
