# Supplementary material for: Extensive horizontal gene transfers between plant pathogenic fungi
Source: BMC Biol. 2016 May 23;14:41. doi: 10.1186/s12915-016-0264-3 (PMC4876562; doi:10.1186/s12915-016-0264-3)
Supplement: Additional file 2: — Pezizomycotina RefSeq proteomic data included in the two-way phylogenomic analysis in this study. (PDF 157 kb) [file 12915_2016_264_MOESM2_ESM.pdf]

Additional file 2. Pezizomycotina RefSeq proteomic data included in the 2-way phylogenomic analysis in this study. The 22 species shown in the red text are Sordariomycetes species that are included in the tree construction (Fig. 1B and Additional file 1).

| No. | Class | Order | Species | NCBI Genome ID* |
|---|---|---|---|---|
| 1 | Sordariomycetes | Magnaporthales-CladeB | *Pyricularia grisea* | [1] |
| 2 | Sordariomycetes | Magnaporthales-CladeB | *Macgarvieomyces juncicola* | [1] |
| 3 | Sordariomycetes | Magnaporthales-CladeB | ***Pyricularia oryzae*** | 62 |
| 4 | Sordariomycetes | Magnaporthales-CladeB | *Xenopyricularia zizaniicola* | [1] |
| 5 | Sordariomycetes | Magnaporthales-CladeC | *Buergenerula spartinae* | [1] |
| 6 | Sordariomycetes | Magnaporthales-CladeC | *Slopeiomyces cylindrosporus* | [1] |
| 7 | Sordariomycetes | Magnaporthales-CladeC | *Gaeumannomyces graminis* | [1] |
| 8 | Sordariomycetes | Magnaporthales-CladeC | *Nakataea oryzae* | [1] |
| 9 | Sordariomycetes | Magnaporthales-CladeC | ***Magnaporthiopsis incrustans*** | [1] |
| 10 | Sordariomycetes | Magnaporthales-CladeC | *Magnaporthiopsis panicorum* | [1] |
| 11 | Sordariomycetes | Magnaporthales-CladeC | *Magnaporthiopsis rhizophila* | [1] |
| 12 | Sordariomycetes | Magnaporthales-CladeC | *Omnidemptus affinis* | [1] |
| 13 | Sordariomycetes | Magnaporthales-CladeC | *Pseudophialophora eragrostis* | [1] |
| 14 | Sordariomycetes | Magnaporthales-CladeC | *Pseudophialophora panicorum* | [1] |
| 15 | Sordariomycetes | Magnaporthales-CladeC | *Pseudophialophora schizachyrii* | [1] |
| 16 | Sordariomycetes | Magnaporthales-CladeC | *Bussabanomyces longisporus* | [1] |
| 17 | Sordariomycetes | Magnaporthales-CladeA | *Ophioceras commune* | [1] |
| 18 | Sordariomycetes | Magnaporthales-CladeA | ***Ophioceras dolichostomum*** | [1] |
| 19 | Sordariomycetes | Magnaporthales-CladeA | *Ophioceras leptosporum* | [1] |
| 20 | Sordariomycetes | Magnaporthales-CladeA | ***Pseudohelonectia lignicola*** | [1] |
| 21 | Sordariomycetes | Diaporthales | *Cryphonectria parasitica* | Crypa1 (JGI) |
| 22 | Sordariomycetes | Diaporthales | ***Diaporthe ampelina*** | 38176 |
| 23 | Sordariomycetes | Diaporthales | *Diaporthe longicolla* | 14992 |
| 24 | Sordariomycetes | Diaporthales | ***Togninia minima (P. aleophilum)*** | [2] |
| 25 | Sordariomycetes | Diaporthales | *Valsa mali* | 35576 |
| 26 | Sordariomycetes | Ophiostomatales | ***Grosmannia clavigera*** | 2302 |
| 27 | Sordariomycetes | Ophiostomatales | ***Ophiostoma piceae*** | 18073 |
| 28 | Sordariomycetes | Ophiostomatales | *Ophiostoma ulmi* | [3] |
| 29 | Sordariomycetes | Ophiostomatales | ***Sporothrix brasiliensis*** | 35789 |
| 30 | Sordariomycetes | Ophiostomatales | *Sporothrix schenckii* | 22522 |
| 31 | Sordariomycetes | Hypocreales | *Beauveria bassiana* | 910 |
| 32 | Sordariomycetes | Hypocreales | *Cordyceps militaris* | 10687 |
| 33 | Sordariomycetes | Hypocreales | *Fusarium graminearum* | 58 |
| 34 | Sordariomycetes | Hypocreales | *Fusarium pseudograminearum* | 14399 |
| 35 | Sordariomycetes | Hypocreales | ***Nectria haematococca (F. solani)*** | 537 |
| 36 | Sordariomycetes | Hypocreales | *Metarhizium acridum* | 2443 |
| 37 | Sordariomycetes | Hypocreales | *Metarhizium majus* | 37185 |
| 38 | Sordariomycetes | Hypocreales | *Metarhizium robertsii* | 13329 |
| 39 | Sordariomycetes | Hypocreales | ***Trichoderma reesei*** | 323 |
| 40 | Sordariomycetes | Hypocreales | *Verticillium alfalfae* | 21497 |
| 41 | Sordariomycetes | Hypocreales | ***Verticillium dahliae*** | 832 |
| 42 | Sordariomycetes | Hypocreales | *Verticillium tricorpus* | 32535 |
| 43 | Sordariomycetes | Sordariales | *Chaetomium globosum* | 304 |
| 44 | Sordariomycetes | Sordariales | *Chaetomium thermophilum* | 2820 |
| 45 | Sordariomycetes | Sordariales | ***Myceliophthora thermophila*** | 10695 |
| 46 | Sordariomycetes | Sordariales | ***Neurospora crassa*** | 19 |
| 47 | Sordariomycetes | Sordariales | *Neurospora tetrasperma* | 3646 |
| 48 | Sordariomycetes | Sordariales | ***Podospora anserina*** | [4] |
| 49 | Sordariomycetes | Sordariales | *Sordaria macrospora* | 2242 |
| 50 | Sordariomycetes | Sordariales | *Thielavia terrestris* | 10696 |
| 51 | Sordariomycetes | Glomerellales | ***Colletotrichum fioriniae*** | 17405 |
| 52 | Sordariomycetes | Glomerellales | ***Colletotrichum gloeosporioides*** | 17739 |
| 53 | Sordariomycetes | Glomerellales | ***Colletotrichum graminicola*** | 2138 |
| 54 | Sordariomycetes | Glomerellales | *Colletotrichum higginsianum* | 11306 |
| 55 | Sordariomycetes | Glomerellales | ***Colletotrichum orbiculare*** | 15316 |
| 56 | Sordariomycetes | Glomerellales | *Colletotrichum sublineola* | 32004 |

| | | | | |
|---|---|---|---|---|
| 57 | Dothideomycetes | Capnodiales | *Baudoinia panamericana* | 40216 |
| 58 | Dothideomycetes | Pleosporales | *Bipolaris oryzae* | 13435 |
| 59 | Dothideomycetes | Pleosporales | *Bipolaris sorokiniana* | 3236 |
| 60 | Dothideomycetes | Pleosporales | *Bipolaris zeicola* | 13436 |
| 61 | Dothideomycetes | Pleosporales | *Leptosphaeria maculans* | Genbank:GCA_000230375.1 |
| 62 | Dothideomycetes | Botryosphaeriales | *Neofusicoccum parvum* | 16686 |
| 63 | Dothideomycetes | Pleosporales | *Phaeosphaeria nodorum* | 456 |
| 64 | Dothideomycetes | Capnodiales | *Pseudocercospora fijiensis* | GenBank:AIHZ00000000.1 |
| 65 | Dothideomycetes | Pleosporales | *Pyrenophora teres* | 2995 |
| 66 | Dothideomycetes | Pleosporales | *Pyrenophora tritici* | 706 |
| 67 | Dothideomycetes | Pleosporales | *Setosphaeria turcica* | 12532 |
| 68 | Dothideomycetes | Incertae sedis | *Zymoseptoria tritici* | 454 |
| 69 | Eurotiomycetes | Onygenales | *Arthroderma benhamiae* | 887 |
| 70 | Eurotiomycetes | Onygenales | *Arthroderma otae* | 796 |
| 71 | Eurotiomycetes | Eurotiales | *Aspergillus clavatus* | 499 |
| 72 | Eurotiomycetes | Eurotiales | *Aspergillus flavus* | 360 |
| 73 | Eurotiomycetes | Eurotiales | *Aspergillus fumigatus* | 18 |
| 74 | Eurotiomycetes | Eurotiales | *Aspergillus nidulans* | 17 |
| 75 | Eurotiomycetes | Eurotiales | *Aspergillus niger* | 429 |
| 76 | Eurotiomycetes | Eurotiales | *Aspergillus oryzae* | 526 |
| 77 | Eurotiomycetes | Eurotiales | *Aspergillus terreus* | 53 |
| 78 | Eurotiomycetes | Incertae sedis | *Blastomyces dermatitidis* | 255 |
| 79 | Eurotiomycetes | Chaetothyriales | *Capronia coronata* | 13900 |
| 80 | Eurotiomycetes | Chaetothyriales | *Capronia epimyces* | 13901 |
| 81 | Eurotiomycetes | Chaetothyriales | *Cladophialophora carrionii* | 16215 |
| 82 | Eurotiomycetes | Chaetothyriales | *Cladophialophora psammophila* | 16322 |
| 83 | Eurotiomycetes | Chaetothyriales | *Cladophialophora yegresii* | 13902 |
| 84 | Eurotiomycetes | Onygenales | *Coccidioides immitis* | 317 |
| 85 | Eurotiomycetes | Onygenales | *Coccidioides posadasii* | 88 |
| 86 | Eurotiomycetes | Onygenales | *Coniosporium apollinis* | 13345 |
| 87 | Eurotiomycetes | Chaetothyriales | *Cyphellophora europaea* | 14776 |
| 88 | Eurotiomycetes | Verrucariales | *Endocarpon pusillum* | 24250 |
| 89 | Eurotiomycetes | Chaetothyriales | *Exophiala dermatitidis* | 2962 |
| 90 | Eurotiomycetes | Onygenales | ***Histoplasma capsulatum*** | 243 |
| 91 | Eurotiomycetes | Onygenales | *Microsporum gypseum* | 800 |
| 92 | Eurotiomycetes | Eurotiales | *Neosartorya fischeri* | 500 |
| 93 | Eurotiomycetes | Onygenales | *Paracoccidioides brasiliensis* | 334 |
| 94 | Eurotiomycetes | Onygenales | *Paracoccidioides* sp. lutzii | 15356 |
| 95 | Eurotiomycetes | Eurotiales | *Penicillium rubens* | [5] |
| 96 | Eurotiomycetes | Eurotiales | *Talaromyces marneffei* | 728 |
| 97 | Eurotiomycetes | Eurotiales | *Talaromyces stipitatus* | 727 |
| 98 | Eurotiomycetes | Onygenales | *Trichophyton rubrum* | 799 |
| 99 | Eurotiomycetes | Onygenales | *Trichophyton verrucosum* | 2293 |
| 100 | Eurotiomycetes | Onygenales | *Uncinocarpus reesii* | 324 |
| 101 | Lecanoromycetes | Lecanorales | ***Cladonia grayi*** | Clagr2 (JGI) |
| 102 | Leotiomycetes | Erysiphales | *Blumeria graminis* | 845 |
| 103 | Leotiomycetes | Helotiales | ***Botrytis cinerea*** | 494 |
| 104 | Leotiomycetes | Helotiales | *Glarea lozoyensis* | 11704 |
| 105 | Leotiomycetes | Helotiales | *Marssonina brunnea* | 14766 |
| 106 | Leotiomycetes | Helotiales | *Sclerotinia sclerotiorum* | 487 |
| 107 | Orbiliomycetes | Orbiliales | *Dactylellina haptotyla* | 17457 |
| 108 | Pezizomycetes | Pezizales | *Tuber melanosporum* | 2215 |
| 109 | Sordariomycetes | Xylariales | *Eutypa lata* | 16685 |
| 110 | Sordariomycetes | Xylariales | *Pestalotiopsis fici* | 14981 |

* When ID Number is not available in NCBI Genome Database, other source information are provided such as the corresponding publication, Genbank accession number, or JGI genome assemblies.

## References

1. Luo J, Qiu H, Cai G, Wagner NE, Bhattacharya D, Zhang N. **Phylogenomic analysis uncovers the evolutionary history of nutrition and infection mode in rice blast fungus and other Magnaporthales**. *Sci Rep.* 2015; **5**:9448.

2. Blanco-Ulate B, Rolshausen P, Cantu D. **Draft genome sequence of the ascomycete *Phaeoacremonium aleophilum* Strain UCR-PA7, a causal agent of the esca disease complex in grapevines**. *Genome Announc.* 2013; **1**. pii: e00390-13
3. Khoshraftar S, Hung S, Khan S, Gong Y, Tyagi V, Parkinson J et al.. **Sequencing and annotation of the *Ophiostoma ulmi* genome**. *BMC Genomics.* 2013; **14**:162.
4. Espagne E, Lespinet O, Malagnac F, Da Silva C, Jaillon O, Porcel BM, Couloux A, Aury JM, Ségurens B, Poulain J, Anthouard V, Grossetete S, Khalili H, Coppin E, Déquard-Chablat M, Picard M, Contamine V, Arnaise S, Bourdais A, Berteaux-Lecellier V, Gautheret D, de Vries RP, Battaglia E, Coutinho PM, Danchin EG, Henrissat B, Khoury RE, Sainsard-Chanet A, Boivin A, Pinan-Lucarré B, Sellem CH, Debuchy R, Wincker P, Weissenbach J, Silar P. **The genome sequence of the model ascomycete fungus Podospora anserina.** *Genome Biol.* 2008; **9**:R77.
5. van den Berg MA, Albang R, Albermann K, Badger JH, Daran JM, Driessen AJ, Garcia-Estrada C, Fedorova ND, Harris DM, Heijne WH, Joardar V, Kiel JA, Kovalchuk A, Martín JF, Nierman WC, Nijland JG, Pronk JT, Roubos JA, van der Klei IJ, van Peij NN, Veenhuis M, von Döhren H, Wagner C, Wortman J, Bovenberg RA. **Genome sequencing and analysis of the filamentous fungus Penicillium chrysogenum.** *Nat Biotechnol.* 2008; **26**:1161-8.
