# Supplementary material for: Extensive horizontal gene transfers between plant pathogenic fungi
Source: BMC Biol. 2016 May 23;14:41. doi: 10.1186/s12915-016-0264-3 (PMC4876562; doi:10.1186/s12915-016-0264-3)
Supplement: Additional file 3: — Magnaporthiopsis incrustans genes putatively derived from non-Pezizomycotina species via horizontal gene transfer. (PDF 173 kb) [file 12915_2016_264_MOESM3_ESM.pdf]

Additional file 3. *Magnaporthiopsis incrustans* genes putatively derived from non-Pezizomycotina species via HGT.

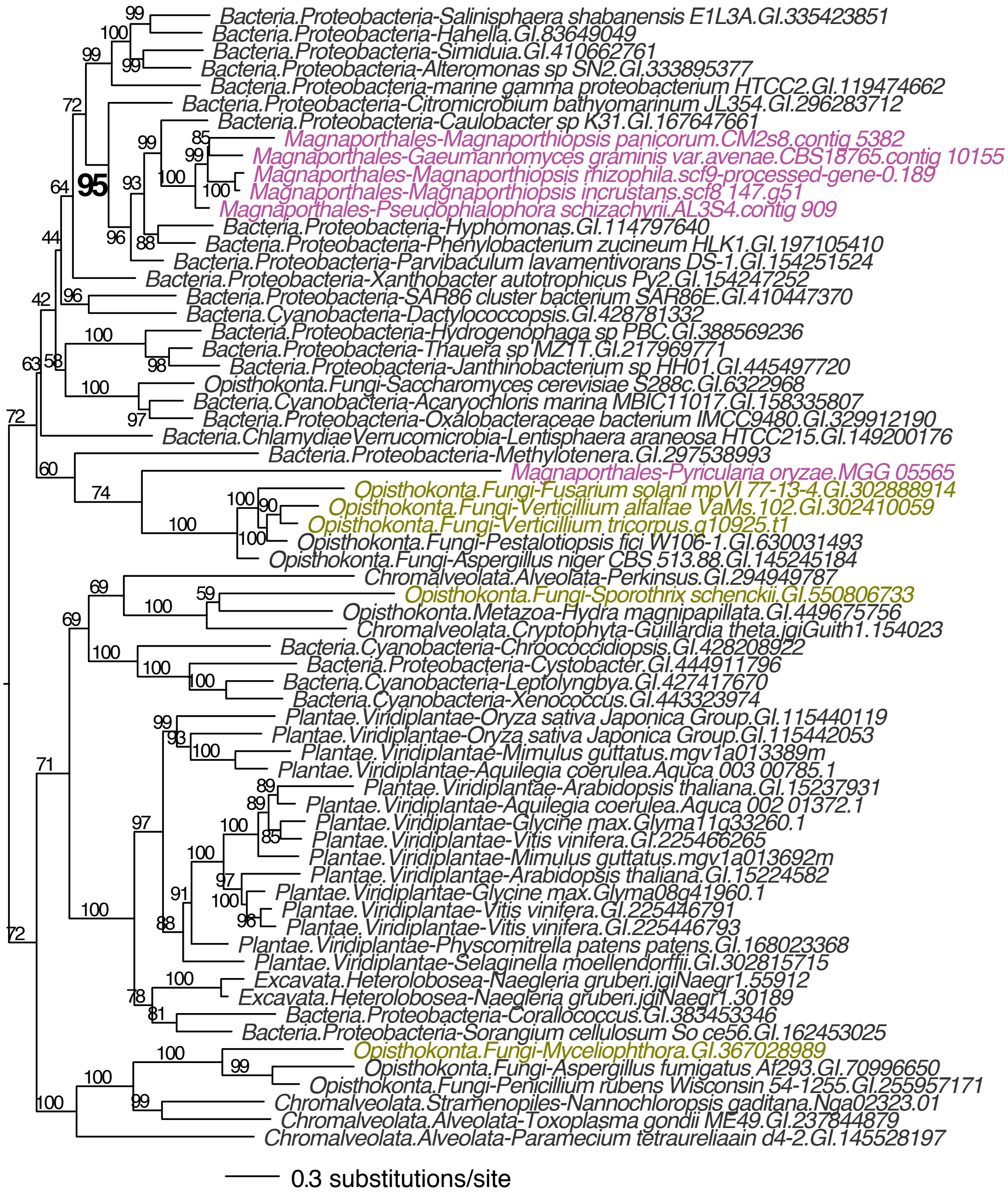

Maximum likelihood tree of a glutathionine S-transferase gene generated by IQtree. Branch supports are UFboot (Ultrafast bootstrap) estimated using 2,000 bootstrap replicates (see Methods in main text). Pink color indicates Magnaporthales. Olive color indicates other Sordariomycetes species.

Additional file 3. *Magnaporthiopsis incrustans* genes putatively derived from non-Pezizomycotina species via HGT.

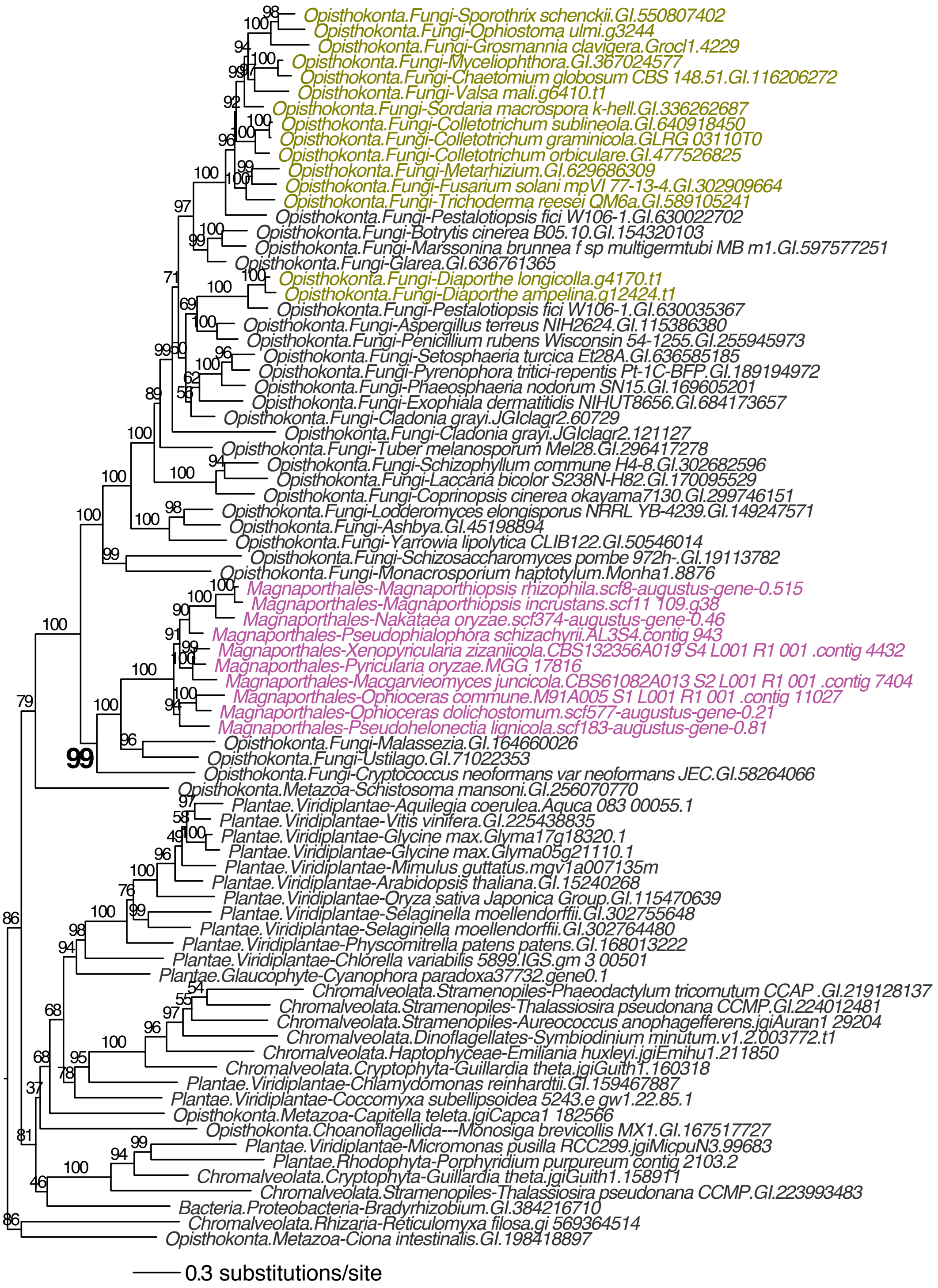

Maximum likelihood tree of a FAD-dependent oxidoreductase gene generated by IQtree. Branch supports are UFboot (Ultrafast boostrap) estimated using 2,000 bootstrap replicates (see Methods in main text). Pink color indicates Magnaporthales. Olive color indicates other Sordariomycetes species.

Additional file 3. *Magnaporthiopsis incrustans* genes putatively derived from non-Pezizomycotina species via HGT.

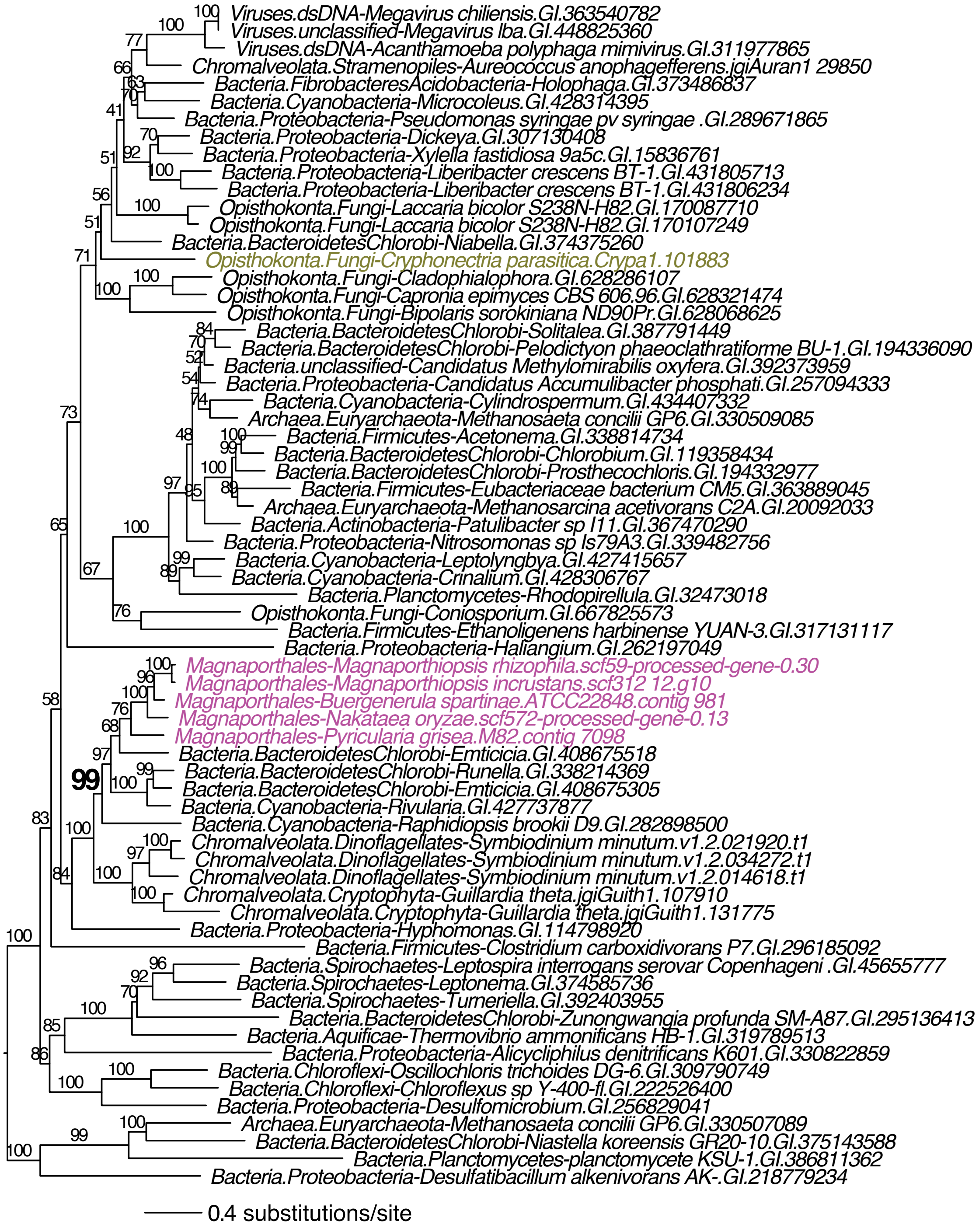

Maximum likelihood tree of a peptidase C1 gene generated by IQtree. Branch supports are UFboot (Ultrafast bootstrap) estimated using 2,000 bootstrap replicates (see Methods in main text). Pink color indicates Magnaporthales. Olive color indicates other Sordariomycetes species.
