# Supplementary material for: Extensive horizontal gene transfers between plant pathogenic fungi
Source: BMC Biol. 2016 May 23;14:41. doi: 10.1186/s12915-016-0264-3 (PMC4876562; doi:10.1186/s12915-016-0264-3)
Supplement: Additional file 4: — The complete set of taxa included in the phylogenetic trees that are shown in Fig. 2 and Figs. 3a and 3b. (PDF 194 kb) [file 12915_2016_264_MOESM4_ESM.pdf]

Additional file 4. The complete set of taxa included in the phylogenetic trees shown in Fig. 2 and Figs. 3A and 3B.

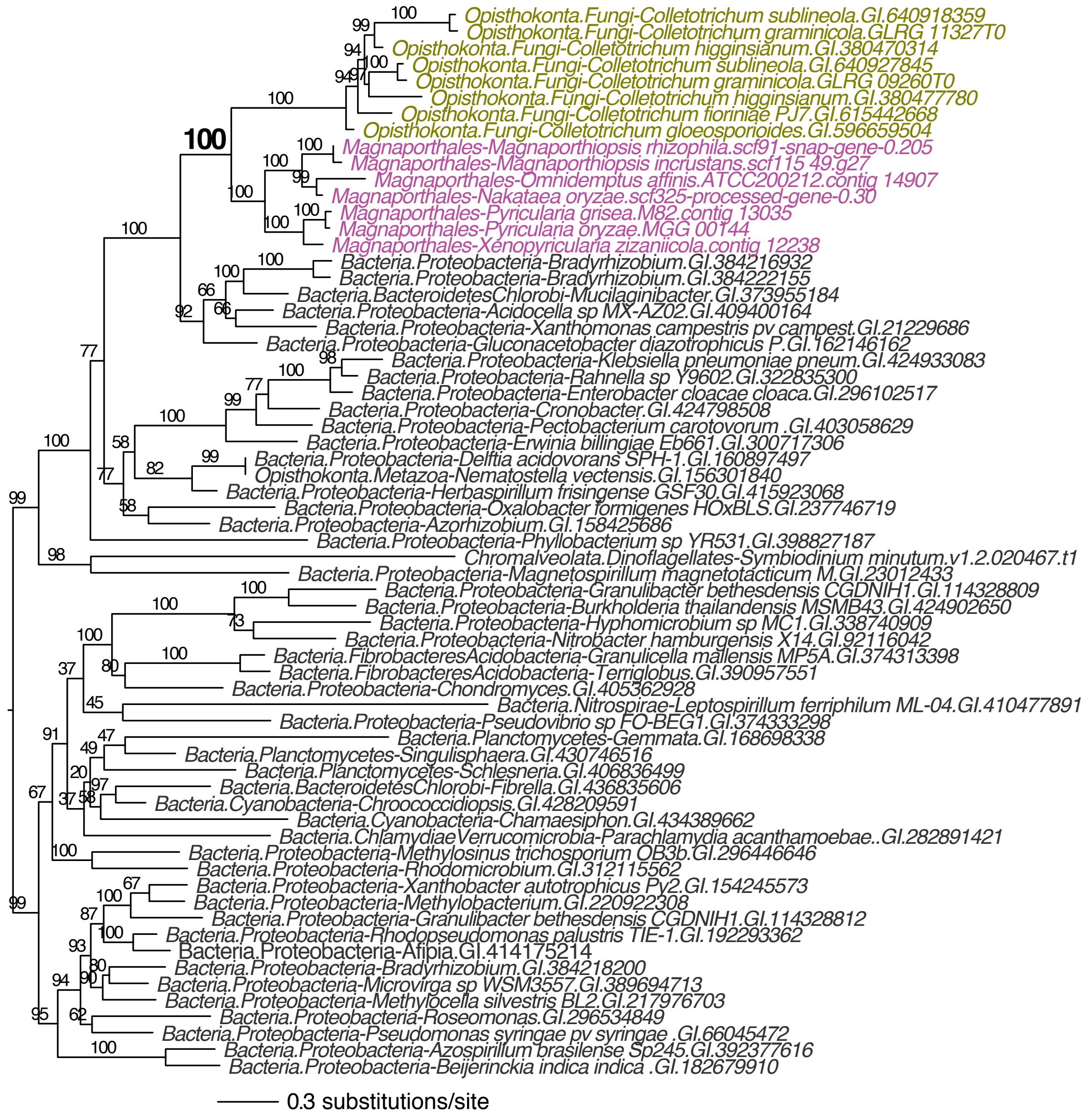

Maximum likelihood tree of a major facilitator superfamily transporter that is shown in Fig. 2A. The tree is built using IQtree and the branch supports are UFboot (Ultrafast bootstrap) estimated using 2,000 bootstrap replicates (see Methods in main text). Pink color indicates Magnaporthales. Olive color indicates *Colletotrichum*. Black color indicates prokaryotic species.

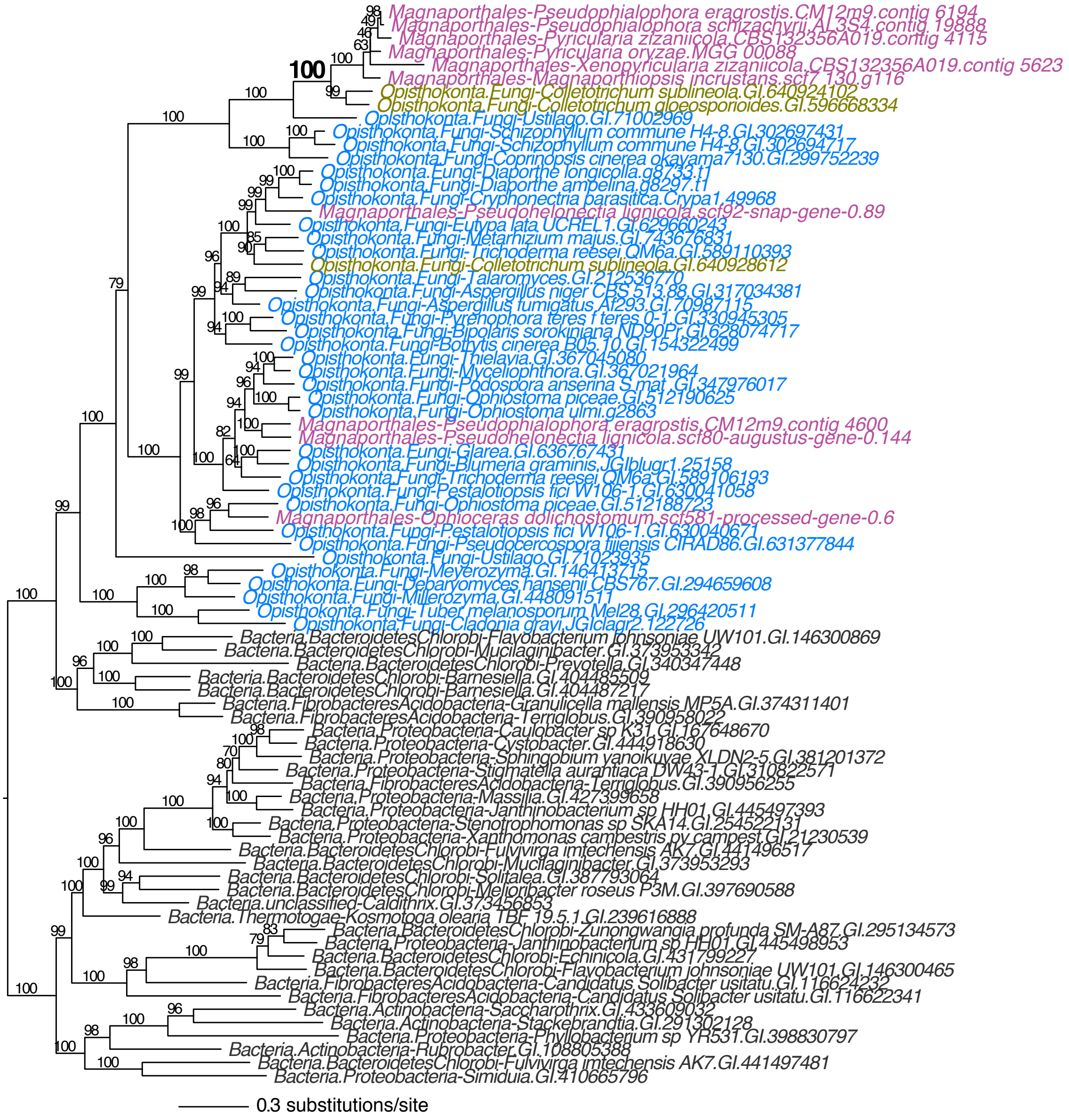

Maximum likelihood tree of a putative alpha-1,2-mannosidase gene that are shown in Fig. 2B. The tree was generated using IQtree and the branch supports are UFboot (Ultrafast boostrap) estimated using 2,000 bootstrap replicates (see Methods in main text). Pink color indicates Magnaporthales. Olive color indicates *Colletotrichum*. Blue color indicates other fungal species. Black color indicates prokaryotic species.

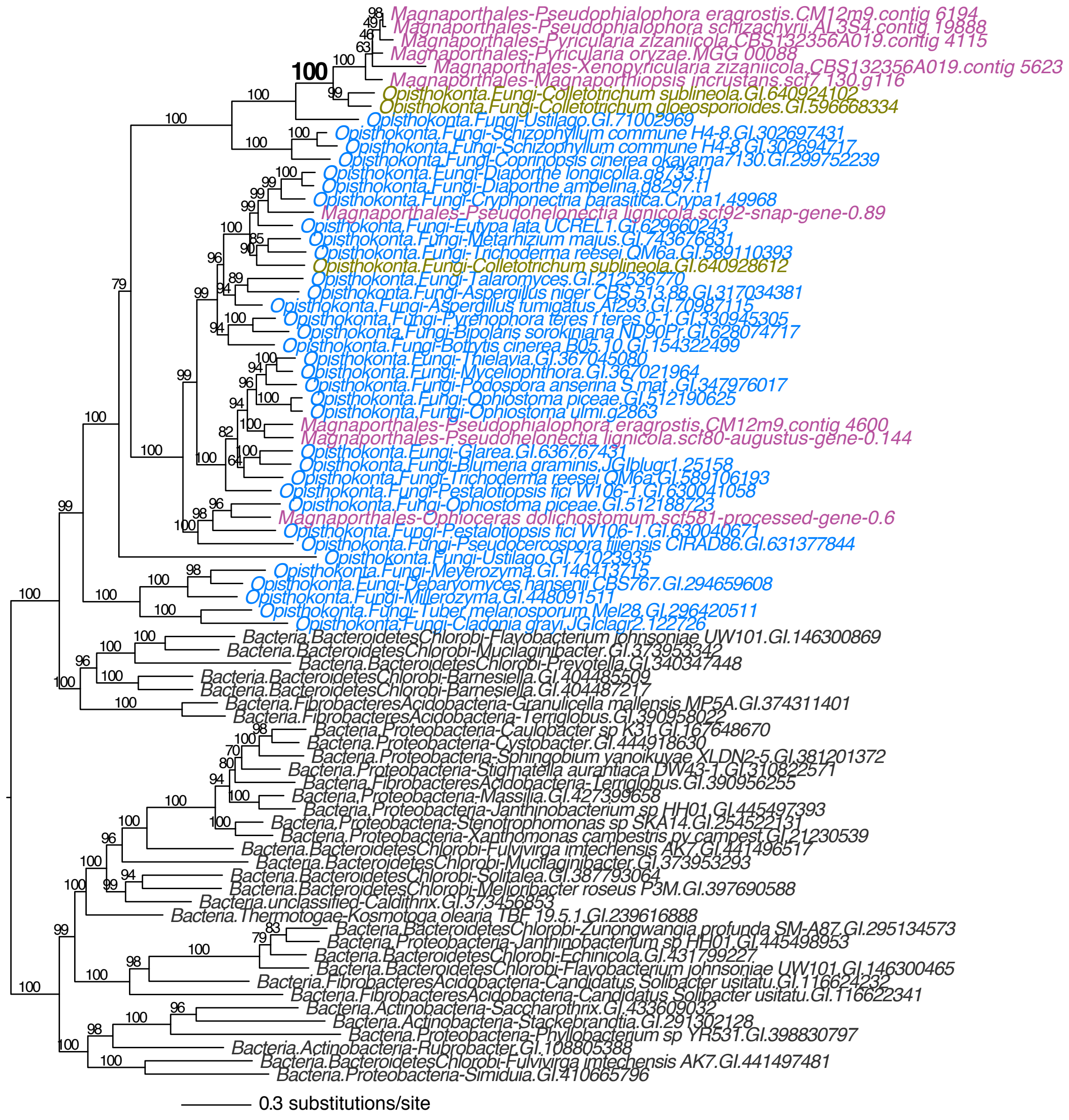

Maximum likelihood tree of a putative dimethylaniline monooxygenase that is shown in Fig. 3A. The tree was generated using IQtree and the branch supports are UFboot (Ultrafast boostrap) estimated using 2,000 bootstrap replicates (see Methods in main text). Pink color indicates Magnaporthales. Olive color indicates *Colletotrichum*. Blue color indicates other fungal species. Black color indicates other species.

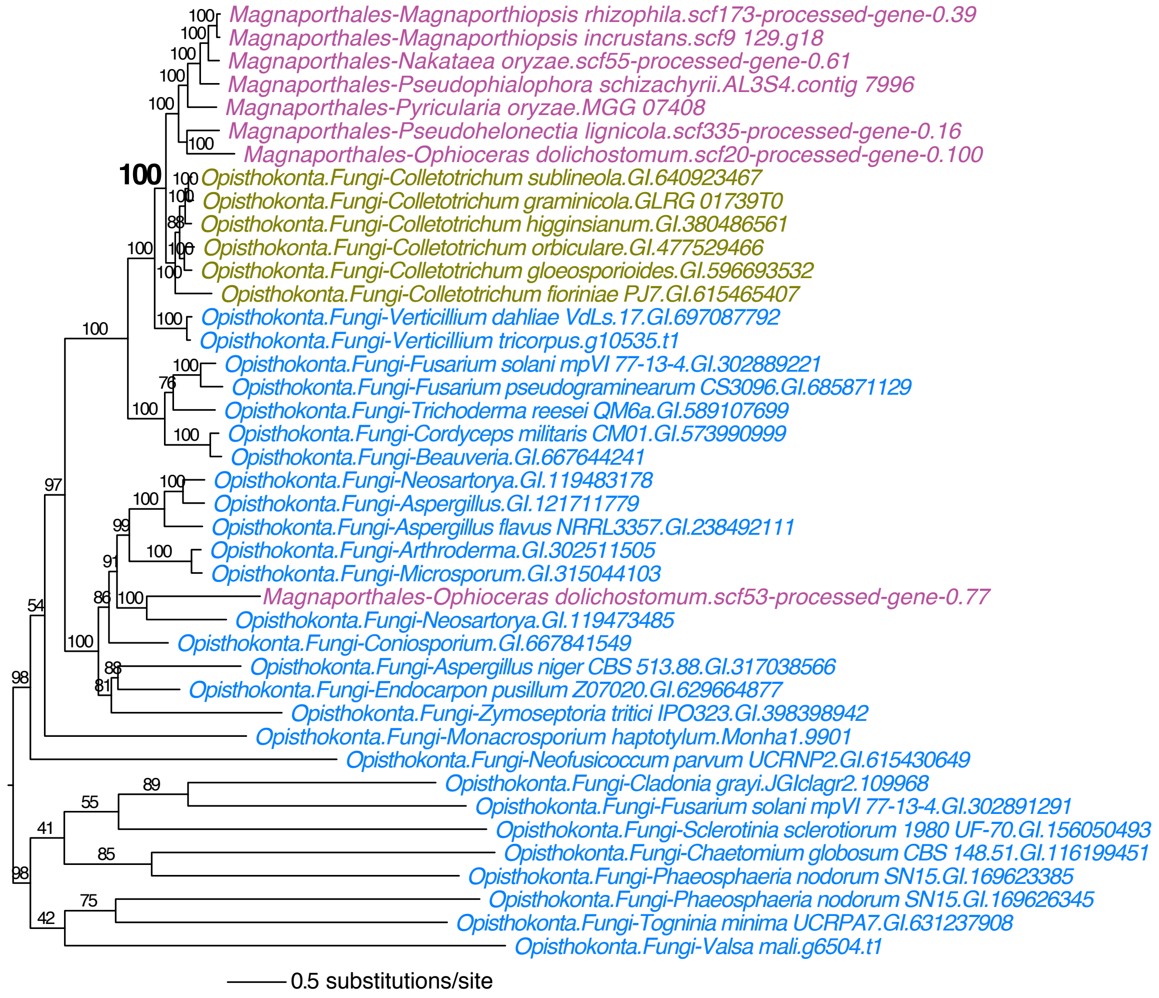

Maximum likelihood tree of a NACHT and TPR domain-containing protein that is shown in Fig. 3B. The tree was generated using IQtree and the branch supports are UFboot (Ultrafast bootstrap) estimated using 2,000 bootstrap replicates (see Methods in main text). Pink color indicates Magnaporthales. Olive color indicates *Colletotrichum*. Blue color indicates other fungal species.
