# Supplementary material for: Extensive horizontal gene transfers between plant pathogenic fungi
Source: BMC Biol. 2016 May 23;14:41. doi: 10.1186/s12915-016-0264-3 (PMC4876562; doi:10.1186/s12915-016-0264-3)
Supplement: Additional file 5: — Annotation of horizontal gene transfers that are transferred between Magnaporthales (using M. incrustans as a reference) and Colletotrichum. (PDF 153 kb) [file 12915_2016_264_MOESM5_ESM.pdf]

Additional file 5. Annotation of HGTs that are transferred between Magnaporthales (using *M. incrustans* as a reference) and *Colletotrichum*

No.	Family	Genes*	Products	UFboot	Mag. Clade[1]	Donor[2]	CAZyme	Transporter	Peptidase	Colleto. long_ctg[3]	No. Colleto.[4]
1	1	*M._incrustans.scf1_204.g77*	arabinan_endo-alpha-l-arabinosidase	100	Clade_C	Magnapo	GH43			1	>1
2	2	*M._incrustans.scf108_47.g25*	ligninase_h2	98	Clade_C	Magnapo	AA2			1	>1
3	3	*M._incrustans.scf11_109.g17*	sodium_symporter_family_protein	100	Clade_C	Colleto				1	>1
4	4	*M._incrustans.scf11_109.g75*	hypothetical_protein_GGTG_03148	92	Clade_ABC	unknown				1	>1
5	5	*M._incrustans.scf113_40.g13*	amino-acid_permease_inda1	100	Clade_A	Magnapo		TC-DB		1	>1
6	6	*M._incrustans.scf113_40.g27*	hypothetical_protein_MGG_02132	100	Clade_ABC	unknown				1	>1
7	7	*M._incrustans.scf114_41.g13*	hypothetical_protein_CFIO01_07988	100	Clade_C	Magnapo				1	1
8	8	*M._incrustans.scf115_49.g24*	tat_pathway_signal_sequence	100	Clade_C	unknown				1	>1
9	9	*M._incrustans.scf115_49.g27*	mfs_transporter	100	Clade_BC	unknown				1	>1
10	10	*M._incrustans.scf135_29.g14*	cation_diffusion_facilitator_1_protein	100	Clade_ABC	unknown				1	>1
11	11	*M._incrustans.scf15_119.g86*	chlorogenic_acid_esterase_precursor	100	Clade_C	Colleto	CE10,CE10		S09X	0	>1
12	12	*M._incrustans.scf163_39.g23*	exoglucanase_3	94	Clade_BC	Magnapo	GH6			1	>1
13	13	*M._incrustans.scf163_39.g5*	hypothetical_protein_GGTG_10533	96	Clade_BC	unknown				1	1
14	14	***M._incrustans.scf18_111.g63***	glycoside_hydrolase	95	Clade_B	Magnapo	CBM18			1	>1
15	15	***M._incrustans.scf18_111.g65***	domain-containing_protein	100	Clade_BC	unknown	CBM50			0	1
16	16	*M._incrustans.scf186_26.g5*	cyclohexanone_-monooxygenase	100	Clade_C	unknown				1	>1
17	17	*M._incrustans.scf19_115.g13*	carboxylesterase_family_protein	100	Clade_C	Magnapo	CE10		S09X	1	>1
18	18	*M._incrustans.scf19_115.g97*	choline_monooxygenase	100	Clade_BC	Colleto				1	>1
19	19	*M._incrustans.scf195_22.g10*	hypothetical_protein_GGTG_04880	100	Clade_C	Magnapo	GH131			1	>1
20	20	***M._incrustans.scf2_209.g41***	hypothetical_protein_GGTG_05571	99	Clade_C	Magnapo	GH115			1	>1
21	21	***M._incrustans.scf2_209.g43***	glycosyl_hydrolase_family_38_domain	100	Clade_BC	Colleto	GH38			1	>1
22	22	*M._incrustans.scf2_209.g60*	arylsulfatase_precursor	100	Clade_C	Magnapo	CBM20|GH15			1	1
23	23	*M._incrustans.scf206_28.g23*	amino_acid_permease	97	Clade_ABC	Colleto		TC-DB		1	>1
24	24	*M._incrustans.scf208_29.g19*	mfs_general_substrate_transporter	100	Clade_C	Magnapo		TC-DB		1	1
25	25	***M._incrustans.scf209_24.g8***	short_chain_dehydrogenase_reductase_family	99	Clade_C	unknown				1	>1
26	26	***M._incrustans.scf209_24.g9***	hypothetical_protein_GGTG_08358	100	Clade_ABC	Colleto				1	>1
27	27	***M._incrustans.scf21_75.g70***	hypothetical_protein_CGGC5_15363	93	Clade_BC	Magnapo				0	1
28	28	***M._incrustans.scf21_75.g71***	methyltransferase_type_partial	100	Clade_C	unknown				0	1
29	29	*M._incrustans.scf225_20.g1*	peptidase_family_m28	100	Clade_ABC	Magnapo	CBM12		M28A	1	>1
30	30	*M._incrustans.scf23_91.g22*	pectate_lyase	99	Clade_BC	Magnapo	PL1,PL1			1	>1
31	31	***M._incrustans.scf233_15.g10***	beta-ketoacyl_synthase_domain-containing	100	Clade_B	Magnapo				1	>1
32		***M._incrustans.scf233_15.g11***		100	Clade_B	Magnapo				1	>1
33	32	***M._incrustans.scf233_15.g12***	enoyl_reductase	100	Clade_B	Magnapo				1	1
34	33	*M._incrustans.scf24_104.g84*	arsenite_efflux_transporter	100	Clade_BC	unknown				1	>1
35	34	*M._incrustans.scf26_101.g7*	acidic_mammalian_chitinase	100	Clade_BC	Magnapo	GH18			1	>1

36	35	*M._incrustans.scf269_12.g7*	hypothetical_protein_GGTG_02130	100	Clade_ABC	unknown				1	>1
37	36	*M._incrustans.scf294_12.g12*	methyltransferase_domain-containing_protein	100	Clade_ABC	Colleto				1	>1
38	37	*M._incrustans.scf3_191.g103*	amino_acid_permease	100	Clade_C	Colleto		TC-DB		1	>1
39	38	*M._incrustans.scf3_191.g97*	nad_-binding_protein	98	Clade_C	unknown				1	>1
40	39	*M._incrustans.scf302_11.g7*	isochorismatase_hydrolase	85	Clade_BC	unknown				1	>1
41	40	*M._incrustans.scf307_9.g9*	mgt_family_glycosyltransferase	99	Clade_BC	Magnapo	GT1			1	>1
42	41	*M._incrustans.scf308_8.g1*	ligninase_lg6_precursor	100	Clade_BC	unknown	AA2			1	>1
43	42	*M._incrustans.scf309_10.g4*	methyltransferase_domain-containing_protein	100	Clade_BC	Colleto				1	>1
44	43	*M._incrustans.scf31_87.g11*	hexose_transporter	99	Clade_BC	Colleto				1	>1
45	44	*M._incrustans.scf31_87.g44*	extracellular_cellulase	95	Clade_ABC	unknown	CBM63			1	>1
46	45	*M._incrustans.scf32_89.g43*	tannase_and_feruloyl_esterase	100	Clade_C	Magnapo				1	>1
47	46	*M._incrustans.scf32_89.g81*	glycosyl_hydrolase_family_81	100	Clade_C	Magnapo	GH81			1	>1
48		*M._incrustans.scf75_46.g31*		100	Clade_C	Colleto	GH81			1	>1
49	47	*M._incrustans.scf321_10.g5*	hypothetical_protein_GGTG_13171	100	Clade_BC	Magnapo				1	>1
50	48	*M._incrustans.scf33_97.g79*	hypothetical_protein_GGTG_05928	100	Clade_ABC	unknown				1	1
51	49	*M._incrustans.scf33_97.g84*	monocarboxylate_permease-like_protein	100	Clade_ABC	Colleto		TC-DB		1	>1
52	50	*M._incrustans.scf34_78.g16*	cornichon_protein	94	Clade_ABC	unknown				1	>1
53	51	*M._incrustans.scf4_208.g109*	choline_transport	96	unclear	Magnapo		TC-DB		1	>1
54	52	*M._incrustans.scf4_208.g112*	dimethylaniline_monooxygenase	100	Clade_C	Magnapo				1	>1
55	53	*M._incrustans.scf4_208.g135*	mannitol_1-phosphate_dehydrogenase	100	Clade_ABC	Magnapo				1	>1
56	54	*M._incrustans.scf4_208.g23*	heterokaryon_incompatibility_protein	100	Clade_C	Colleto				1	>1
57	55	*M._incrustans.scf4_208.g53*	major_facilitator_superfamily_transporter	100	Clade_C	Magnapo				1	>1
58	56	*M._incrustans.scf4_208.g57*	glyoxalase bleomycin_resistance_protein dioxygenase superfamily	100	Clade_C	Colleto				1	>1
59		*M._incrustans.scf46_67.g47*		100	Clade_BC	unknown				1	>1
60	57	*M._incrustans.scf4_208.g79*	asparagine_synthase	100	unclear	Magnapo			C44	1	>1
61	58	*M._incrustans.scf40_91.g81*	tripeptidyl-peptidase_sed4	100	Clade_ABC	Magnapo			S53	1	>1
62	59	*M._incrustans.scf43_84.g22*	leucoanthocyanidin_reductase	99	Clade_BC	Magnapo	CE12			1	>1
63	60	*M._incrustans.scf50_60.g58*	glucose_transporter_hxt1	100	Clade_BC	Colleto				1	>1
64	61	*M._incrustans.scf54_86.g81*	arylsulfatase_protein	100	Clade_B	unknown	CBM20|GH15			0	1
65	62	*M._incrustans.scf55_68.g61*	fatty-acid_amide_hydrolase	100	Clade_B	unknown				1	>1
66	63	*M._incrustans.scf55_68.g64*	major_facilitator_superfamily_transporter	99	Clade_BC	unknown		TC-DB		1	>1
67	64	***M._incrustans.scf61_80.g17***	mfs_multidrug_transporter	100	Clade_BC	Magnapo				1	>1
68	65	***M._incrustans.scf61_80.g18***	c6_finger_domain-containing_protein	100	Clade_BC	Magnapo				1	>1
69	66	*M._incrustans.scf61_80.g74*	glycoside_hydrolase_family_43_protein	96	Clade_C	Magnapo				1	>1
70	67	*M._incrustans.scf63_70.g23*	endoplasmic_reticulum_protein	99	Clade_ABC	Magnapo				1	>1
71	68	*M._incrustans.scf65_53.g1*	ankyrin_repeat-containing	100	Clade_C	unknown				0	1
72	69	*M._incrustans.scf65_53.g44*	mas3_protein	89	Clade_BC	Magnapo				1	>1
73	70	*M._incrustans.scf66_71.g16*	general_amino_acid_permease_agp2	100	Clade_BC	Colleto		TC-DB		1	>1
74	71	*M._incrustans.scf68_59.g21*	nitroreductase_family_protein	100	Clade_A	Magnapo				1	1
75	72	***M._incrustans.scf7_130.g116***	glycosyl_hydrolase	100	Clade_BC	Magnapo	GH92			1	>1
76	73	***M._incrustans.scf7_130.g118***	sulphydryl_oxidase	100	Clade_B	Magnapo				1	>1

77	74	*M._incrustans.scf75_46.g36*	glycosyltransferase_family_90_protein	100	Clade_BC	Magnapo	GT90			1	1
78		*M._incrustans.scf83_65.g53*		100	Clade_ABC	Magnapo	GT90			1	1
79	75	*M._incrustans.scf8_147.g137*	polyketide_synthase	100	Clade_ABC	unknown	GT2			1	>1
80	76	*M._incrustans.scf8_147.g65*	abc_transporter	100	Clade_ABC	Magnapo				1	>1
81	77	*M._incrustans.scf8_147.g9*	secreted_glucosidase	100	Clade_BC	Magnapo	GH16			1	>1
82	78	*M._incrustans.scf81_37.g22*	rhamnogalacturonan_acetylesterase	100	Clade_A	unknown	CE12			1	>1
83	79	*M._incrustans.scf83_65.g47*	choline_dehydrogenase	100	Clade_ABC	Colleto	AA3			1	>1
84	80	***M._incrustans.scf86_70.g25***	aminotransferase_class_i_and_ii	100	Clade_B	unknown				1	1
85	81	***M._incrustans.scf86_70.g26***	epoxide_hydrolase_2	100	Clade_B	Magnapo			S33	1	1
86	82	***M._incrustans.scf86_70.g27***	cytochrome_p450	100	Clade_B	Magnapo				1	1
87	83	*M._incrustans.scf9_129.g18*	nacht_and_tpr_domain-containing_protein	99	Clade_ABC	Colleto				1	>1
88	84	*M._incrustans.scf9_129.g28*	glycoside_hydrolase_family_18_protein	98	Clade_C	unknown	CBM18			0	1
89	85	*M._incrustans.scf9_129.g61*	calcium-transporting_atpase	97	Clade_ABC	Colleto		TC-DB		1	>1
90	86	*M._incrustans.scf91_52.g17*	hypothetical_protein_GGTG_06400	94	Clade_BC	unknown				1	>1
91	87	*M._incrustans.scf94_48.g2*	high-affinity_nicotinic_acid_transporter	99	Clade_ABC	unknown		TC-DB		1	>1
92	88	*M._incrustans.scf94_48.g8*	major_facilitator_superfamily_transporter	99	Clade_BC	Magnapo				1	>1
93	89	*M._incrustans.scf97_44.g2*	phospholipase_carboxylesterase	94	Clade_B	Magnapo				1	>1

* Genes in transferred genomic segments are shown in bold face.

[1] Magnaporthales comprise three major clades (i.e., clades A, B and C). This column indicates tentatively which Magnaporthales clades were involved in the gene transfer with *Colletotrichum*.

[2] The putative donor of the gene transfers between Magnaporthales and *Colletotrichum*.

[3] If one or more transferred *Colletotrichum* genes are located in long contigs (encoding ≥5 genes), value (1) is assigned. Value (0) is assigned otherwise.

[4] Number of *Colletotrichum* species involved in the individual HGT instance.
