# Supplementary material for: Extensive horizontal gene transfers between plant pathogenic fungi
Source: BMC Biol. 2016 May 23;14:41. doi: 10.1186/s12915-016-0264-3 (PMC4876562; doi:10.1186/s12915-016-0264-3)
Supplement: Additional file 6: — Phylogenies of two physically linked genes that were transferred as a genomic segment between Magnaporthales and Colletotrichum. (PDF 105 kb) [file 12915_2016_264_MOESM6_ESM.pdf]

Additional file 6. Phylogenies of two physically linked genes that were transferred as a genomic segment between Magnaporthales and *Colletotrichum*.

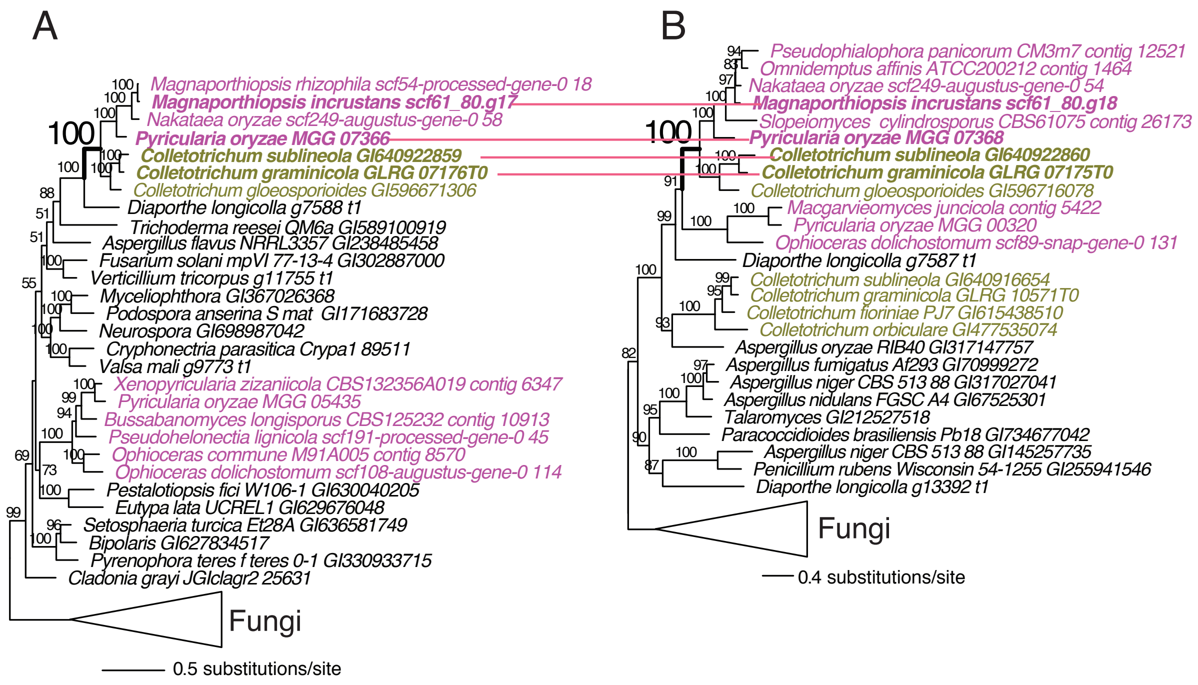

Maximum likelihood trees of two genes sitting side by side in Magnaporthales (e.g., *M. incrustans* and *P. oryzae*) and in *Colletotrichum* (e.g., *C. sublineola* and *C. graminicola*) genomes. (A) ML tree of a major facilitator superfamily protein. (B) ML tree of a putative membrane protein. Red horizontal lines indicate genes from same species. The physical linkages of the genes are reflected by their ID numbers such as scf61_80.g17-g18 in *M. incrustans* and GLRG_07176T0-5T0 in *C. graminicola*. Branch supports are UFboot (Ultrafast boostrap) estimated using 2,000 bootstrap replicates (see Methods in main text for details). The complete tree files are provided in Additional file 11. Pink color indicates Magnaporthales. Olive color indicates *Colletotrichum*.
