# Supplementary material for: Extensive horizontal gene transfers between plant pathogenic fungi
Source: BMC Biol. 2016 May 23;14:41. doi: 10.1186/s12915-016-0264-3 (PMC4876562; doi:10.1186/s12915-016-0264-3)
Supplement: Additional file 7: — Horizontal gene transfer analysis of Ophioceras dolichostomum data. (PDF 101 kb) [file 12915_2016_264_MOESM7_ESM.pdf]

Additional file 7. HGT analysis of *Ophioceras dolichostomum* genome data.

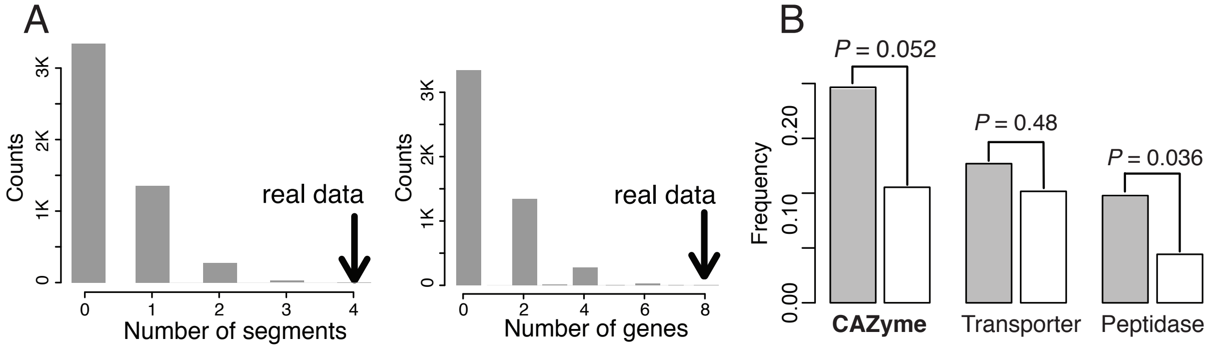

These results were used to corroborate the findings presented in Figs. 3C and 3D. (A) Random sampling analysis of genomic segments in *O. dolichostomum* with regard to the HGT findings. We randomly sampled 51 genes from the *O. dolichostomum* data 5,000 times (see Methods) and the number of genomic segments derived from these replicates (represented by the histogram) ranged from 0-4. In >99.9% (4,955) of the replicates, ≤3 genomic segments were returned. Therefore, the chance is <0.1% to generate four genomic segments that are observed in the actual data (the thick black arrow). Similarly, the entire range of the genes that were included in the genomic segments was 0-8 with >99.9% of the gene numbers being ≤6. Therefore, the chance is <0.1% to generate a total of 8 genes that are contained in genomic segments. These results suggest the enrichment of physical linkage in our HGT data that cannot be explained by random chance alone. (B) The proportions of CAZymes, transporters, and peptidases among the transferred genes (gray color) compared to those in complete-genome data (white color). The statistical significance is shown for each comparison.
