# Supplementary material for: Extensive horizontal gene transfers between plant pathogenic fungi
Source: BMC Biol. 2016 May 23;14:41. doi: 10.1186/s12915-016-0264-3 (PMC4876562; doi:10.1186/s12915-016-0264-3)
Supplement: Additional file 8: — Annotation of horizontal gene transfers that are transferred between Magnaporthales (using O. dolichostomum as a reference) and Colletotrichum. (PDF 140 kb) [file 12915_2016_264_MOESM8_ESM.pdf]

Additional file 8. Annotation of HGTs that are transferred between Magnaporthales (using *O. dolichostomum* as a reference) and *Colletotrichum*

No.	Family	Genes*	Products	UFboot	Mag. Clade [1]	Donor[2]	CAZyme	Transporter	Peptidase	Colleto. long_contig[3]	No. Colleto.[4]
1	1	*O._dolichostomum.scf101_28.g24*	hypothetical_protein_UCRPA7_6765	88	Clade_ABC	unknown				1	>1
2	2	*O._dolichostomum.scf11_77.g11*	cellulose_-beta-cellobiosidase	100	Clade_ABC	Colleto	GH7			1	>1
3	3	*O._dolichostomum.scf126_25.g16*	mas3_protein	85	Clade_ABC	Magnapo				1	>1
4	4	*O._dolichostomum.scf144_30.g6*	heterokaryon_incompatibility_protein	96	Clade_A	unknown				1	>1
5	5	*O._dolichostomum.scf151_30.g15*	endoplasmic_reticulum_protein	99	Clade_ABC	Magnapo				1	>1
6	6	*O._dolichostomum.scf154_22.g13*	extracellular_dihydrogeodin_oxidase	100	Clade_ABC	unknown	AA1	TC-DB		1	>1
7	7	*O._dolichostomum.scf166_20.g2*	dj-1_family_protein	99	Clade_ABC	unknown			C56	1	>1
8	8	*O._dolichostomum.scf188_26.g8*	peroxysomal_citrate_synthase	100	Clade_A	Colleto				1	>1
9	9	*O._dolichostomum.scf190_26.g6*	hypothetical_protein	100	Clade_ABC	Colleto				1	>1
10	10	*O._dolichostomum.scf20_68.g26*	nacht_and_tpr_domain-containing	98	Clade_ABC	Colleto				1	>1
11	11	***O._dolichostomum.scf201_21.g1***	hypothetical_protein	100	Clade_ABC	unknown				1	>1
12	12	***O._dolichostomum.scf201_21.g3***	delta_-sterol_reductase	99	Clade_ABC	Magnapo				1	>1
13	13	***O._dolichostomum.scf202_21.g15***	hypothetical_protein	100	Clade_ABC	unknown				1	1
14	14	***O._dolichostomum.scf202_21.g16***	monocarboxylate_permease-like	100	Clade_ABC	Colleto		TC-DB		1	>1
15	15	*O._dolichostomum.scf209_20.g7*	hypothetical_protein	100	Clade_A	unknown				1	>1
16	16	*O._dolichostomum.scf239_23.g16*	hypothetical_protein	100	Clade_A	unknown			M38	1	1
17	17	***O._dolichostomum.scf278_18.g13***	transcription_factor_domain-containing	100	Clade_A	unknown				1	>1
18	18	***O._dolichostomum.scf278_18.g15***	mannitol_1-phosphate_dehydrogenase	100	Clade_ABC	Magnapo				1	>1
19	19	*O._dolichostomum.scf290_20.g12*	amino-acid_permease_inda1	100	Clade_A	unknown		TC-DB		1	>1
20	20	*O._dolichostomum.scf300_18.g3*	hypothetical_protein	87	Clade_A	Magnapo				1	>1
21	21	*O._dolichostomum.scf304_7.g6*	acyltransferase-like_protein	99	Clade_C	Magnapo				1	1
22	22	*O._dolichostomum.scf33_56.g43*	hypothetical_protein	98	Clade_ABC	unknown				1	1
23	23	*O._dolichostomum.scf357_13.g5*	hypothetical_protein	100	Clade_ABC	Magnapo				1	>1
24	24	*O._dolichostomum.scf381_9.g9*	abc_transporter	100	Clade_ABC	unknown		TC-DB		1	>1
25	25	*O._dolichostomum.scf395_11.g11*	pheromone-regulated_membrane_pro.	96	Clade_ABC	Colleto				1	>1
26	26	***O._dolichostomum.scf4_87.g18***	hypothetical_protein	100	Clade_A	Colleto				1	>1
27	27	***O._dolichostomum.scf4_87.g20***	hypothetical_protein	100	Clade_A	Colleto				1	>1
28	28	*O._dolichostomum.scf405_11.g4*	methyltransferase_domain-containing	100	Clade_ABC	unknown				1	>1
29	29	*O._dolichostomum.scf43_45.g19*	nadph-dependent_fmn_reductase	98	Clade_ABC	unknown				1	>1
30	30	*O._dolichostomum.scf458_9.g5*	hypothetical_protein	100	Clade_ABC	unknown		TC-DB		1	>1
31	31	*O._dolichostomum.scf460_13.g3*	peptidase_family_m28	100	Clade_ABC	Magnapo	CBM12		M28A	1	>1
32	32	*O._dolichostomum.scf464_12.g9*	fungal_cellulose_binding_domain	100	Clade_A	Colleto	GH5|CBM1			1	>1
33	33	*O._dolichostomum.scf49_41.g18*	cation_diffusion_facilitator_1_protein	99	Clade_ABC	unknown				1	>1
34	34	*O._dolichostomum.scf493_7.g4*	serine_carboxypeptidase_s28	100	Clade_ABC	unknown			S28	1	>1
35	35	*O._dolichostomum.scf493_7.g7*	choline_dehydrogenase	100	Clade_ABC	Colleto	AA3			1	>1
36	36	*O._dolichostomum.scf50_54.g15*	glycosyl_hydrolase_family_43	100	Clade_A	unknown	GH43			1	>1
37	37	*O._dolichostomum.scf504_7.g1*	chitinase_1	100	Clade_ABC	Colleto	GH18			1	>1
38	38	*O._dolichostomum.scf517_12.g6*	fad_dependent_oxidoreductase	100	Clade_A	unknown				1	>1
39	39	*O._dolichostomum.scf52_54.g42*	glycoside_hydrolase_family_27_protein	99	Clade_A	Colleto	GH27			1	>1

40	40	*O._dolichostomum.scf522_8.g3*	sugar_transporter	100	Clade_BC	Magnapo				1	>1
41	41	*O._dolichostomum.scf525_9.g5*	hypothetical_protein	100	Clade_A	Colleto				1	>1
42	42	*O._dolichostomum.scf53_51.g8*	ncs1_allantoate_transporter_protein	100	Clade_A	unknown		TC-DB		1	>1
43	43	*O._dolichostomum.scf544_8.g7*	serine_carboxypeptidase	100	Clade_A	Magnapo			S6	1	>1
44	44	*O._dolichostomum.scf577_7.g2*	sir2_family_protein	100	Clade_ABC	unknown				1	>1
45	45	*O._dolichostomum.scf578_3.g1*	cellulose_binding_domain-containing	100	Clade_BC	Magnapo	AA3|AA8			1	1
46	46	*O._dolichostomum.scf613_6.g3*	amino_acid_permease	98	Clade_C	Magnapo		TC-DB		1	>1
47	47	*O._dolichostomum.scf658_4.g4*	duf563_domain-containing_protein	86	Clade_ABC	Magnapo	GT61			1	1
48	48	*O._dolichostomum.scf71_46.g2*	alcohol_dehydrogenase	100	Clade_ABC	unknown				1	>1
49	49	*O._dolichostomum.scf81_43.g10*	vesicles_protein_erv14_protein	96	Clade_ABC	unknown				1	>1
50	50	*O._dolichostomum.scf89_37.g18*	thioester_reductase_domain-containing	97	Clade_A	Magnapo				1	>1
51	51	*O._dolichostomum.scf95_39.g35*	tri7-like_toxin_biosynthesis_protein	100	Clade_A	unknown				0	1

* Physically-linked genes are shown in bold face.

[1] Magnaporthales comprise three major clades (i.e., clades A, B and C). This column indicates which clades were involved in the gene transfer with *Colletotrichum*.

[2] The putative donor of the gene transfers between Magnaporthales and *Colletotrichum*.

[3] If one or more transferred *Colletotrichum* genes are located in long contigs (encoding ≥5 genes), value (1) is assigned. Value (0) is assigned otherwise.

[4] Number of *Colletotrichum* species involved in the individual HGT instance.
