# Supplementary material for: Extensive horizontal gene transfers between plant pathogenic fungi
Source: BMC Biol. 2016 May 23;14:41. doi: 10.1186/s12915-016-0264-3 (PMC4876562; doi:10.1186/s12915-016-0264-3)
Supplement: Additional file 9: — Classification of Magnaporthales-Colletotrichum horizontal gene transfers (HGTs) and taxonomic gene mapping and HGT/gene loss counts. (PDF 384 kb) [file 12915_2016_264_MOESM9_ESM.pdf]

Additional file 9. Classification of Magnaporthales-*Colletotrichum* HGTs and taxonomic gene mapping and HGT/gene loss counts

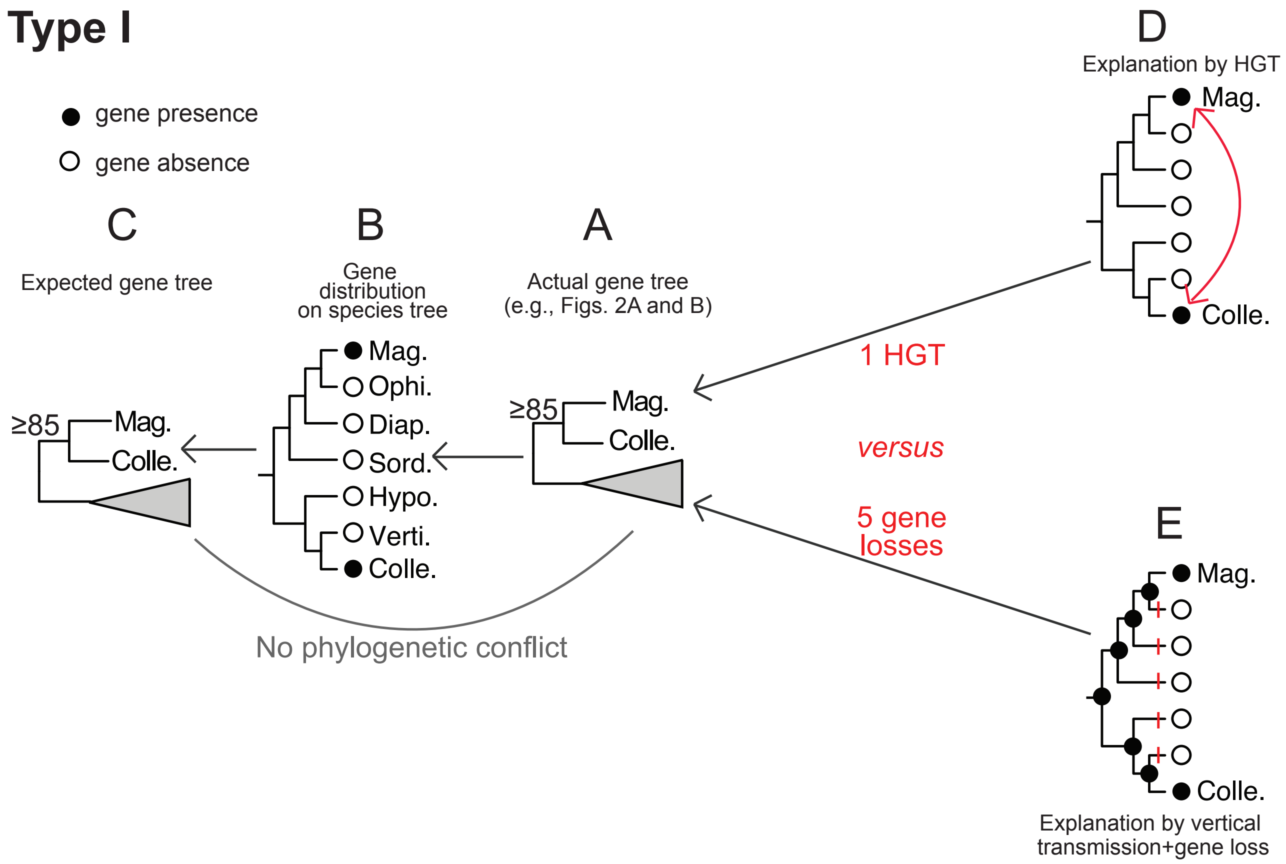

Fig. 1. Taxonomic mapping and HGT/gene loss counts in Type I HGTs. In this category of HGTs, Magnaporthales-*Colletotrichum* forms a sister group relationship (≥85% UFboot support) regardless of the topology and taxonomic composition in the remaining tree (gray triangle in Panel A). When ignoring the remaining tree, the gene is present only in Magnaporthales and *Colletotrichum* in the Sordariomycetes species tree (Panel B, derived from Fig. 1B in main text) and the expected gene tree is the same as the actual gene tree (Panel C). Therefore, no phylogenetic conflict arises in this scenario. To explain the actual gene tree using HGT, a gene is required to be present specifically in Magnaporthales and is then transferred (1 HGT, red arrow in Panel D) into *Colletotrichum*, or the other way round (red arrow in the opposite direction, Panel D). To explain the actual gene tree using vertical inheritance with gene losses, the node uniting Magnaporthales-*Colletotrichum* could be considered to be the Sordariomycetes common ancestor. This gene is lost in all major lineages except Magnaporthales and *Colletotrichum* (Panel E). Therefore, a total of 5 independent gene losses are required (red vertical bars in Panel E). This category requires only Magnaporthales-*Colletotrichum* monophyly and therefore applies to all Magnaporthales-*Colletotrichum* HGT candidates. Magnaporthales (Mag.), Ophiostomatales (Ophi.), Diaporthales (Diap.), Sordariales (Sord.), Hypocreales (Hypo.), *Verticillium* (Verti.), *Colletotrichum* (Colle.).

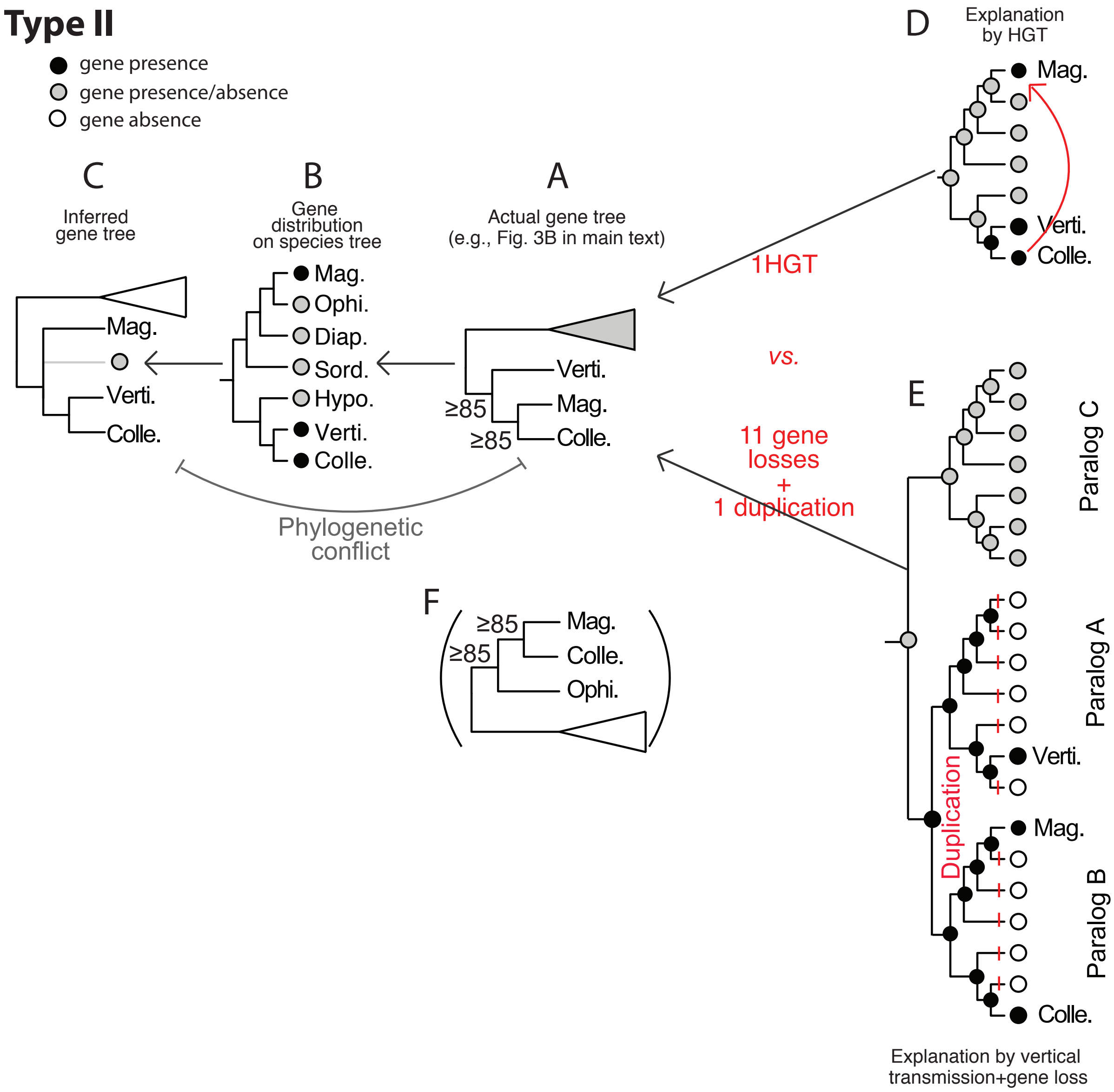

Fig. 2. Taxonomic mapping and phylogenetic conflicts in Type II HGTs. In this category, Magnaporthales-*Colletotrichum* forms a sister group relationship to *Verticillium* (Panel A, see Fig. 3B in main text for an example). The corresponding nodes have ≥85% UFboot supports (Panel A). The remaining tree is collapsed and is represented as a grey triangle (Panel A). In the Sordariomycetes species tree, *Verticillium* and *Colletotrichum* are sister lineage, whereas Magnaporthales correspond to a separate lineage (Panel B, derived from Fig. 1B in main text). The black circles indicate the presence of the genes. Grey circles indicate the presence or absence of the corresponding genes in a case-by-case manner (Panel B). Given the gene distribution in the species tree (Panel B), a generalized gene tree can be inferred (Panel C) with *Verticillium* and *Colletotrichum* in a monophyletic group. Magnaporthales and other Sordariomycetes species (if any, the grey branch and circle) are excluded from the *Verticillium* + *Colletotrichum* monophyletic group (Panel C). Consequently, a phylogenetic conflict arises between the actual gene tree (Panel A) and the inferred gene tree (Panel C). In a scenario of HGT (Panel D), *Verticillium* and *Colletotrichum* inherited the gene from their common ancestor followed by a gene transfer from *Colletotrichum* to Magnaporthales (black circles and the red arrow). Thus, one instance of HGT is sufficient to explain the phylogenetic conflict (Panel D). Gene-presence in other Sordariomycetes species (e.g., Hypocreales such as *Fusarium* in Fig. 3B in main text) and therefore a possible earlier gene origin (grey circles) does not change the conclusion (Panel D).

In a scenario of vertical inheritance and gene loss (Panel E), an instance of gene duplication is required in the Sordariomycetes common ancestor followed by 6 gene losses (red vertical bars) in paralog A leaving genes in only *Verticillium* and 5 gene losses in paralog B leaving genes in only Magnaporthales and *Colletotrichum*. All together, 1 gene duplication and 11 gene losses are required in this second scenario (Panel E). For simplicity, we do not consider the additional gene duplication and gene losses (grey circles and paralog C in Panel E) that may be required in the cases of the gene presence in additional Sordariomycetes species (e.g., Hypocreales such as *Fusarium* in Fig. 3B). Taken together, the changes that are required to explain this category of gene tree (Panel A) are 1 gene transfer assuming HGT *versus* 1 gene duplication + 11 gene losses assuming vertical transmission and gene losses. The same principle applies to gene trees when the Magnaporthales-*Colletotrichum* monophyletic group is sister to Ophiostomatales (Panel F). Ophiostomatales (e.g., *Ophiostoma piceae*) are sister to Magnaporthales (Fig. 1B in main text). Magnaporthales (Mag.), Ophiostomatales (Ophi.), Diaporthales (Diap.), Sordariales (Sord.), Hypocreales (Hypo.), *Verticillium* (Verti.), *Colletotrichum* (Colle.).

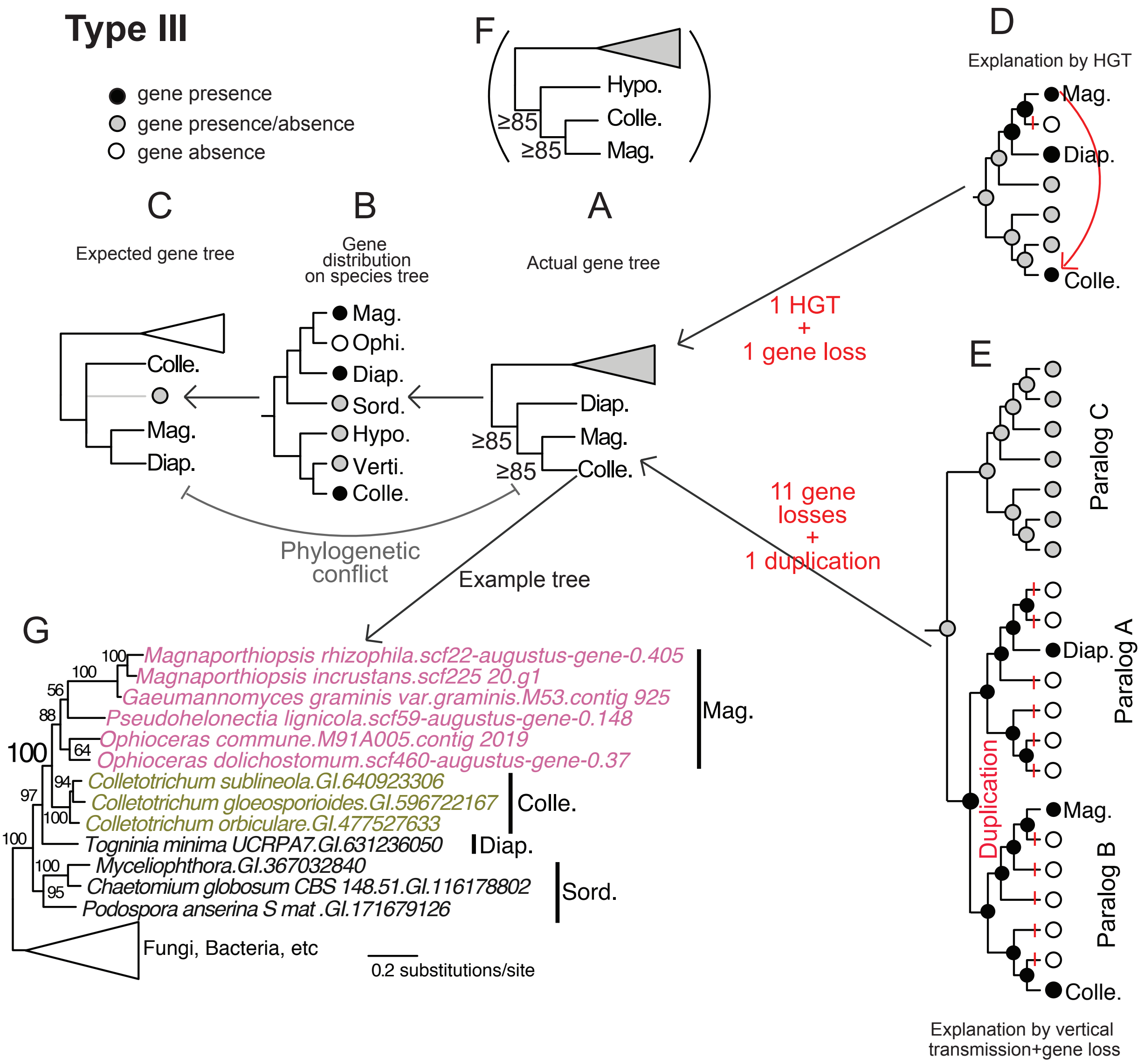

Fig. 3. Taxonomic mapping and phylogenetic conflicts in Type III HGTs. In this category, Magnaporthales-*Colletotrichum* forms a sister group relationship to Diaporthales (e.g., *Tognina minima*) (Panel A, or Panel G for an example). The corresponding nodes have ≥85% UFboot support (Panels A and G). The remaining tree is collapsed and is shown as a grey (Panel A). In the Sordariomycetes species tree, Magnaporthales are more closely related to Diaporthales than to *Colletotrichum* (Panel B, derived from Fig. 1B in main text). The black circles indicate the presence of genes. The open circle indicates gene loss. Grey circles indicate the presence or absence of the corresponding gene in a case-by-case manner (Panel B). Given the gene distribution in the species tree (Panel B), a generalized gene tree can be inferred (Panel C) with Magnaporthales and Diaporthales as a monophyletic group. *Colletotrichum* and other Sordariomycetes species (if any, the grey branch and circle) are excluded from the Magnaporthales + Diaporthales monophyletic group (Panel C). Consequently, a phylogenetic conflict arises between the actual gene tree (Panel A) and the inferred gene tree (Panel C). In a scenario of HGT (Panel D), Magnaporthales and Diaporthales inherit the gene from their common ancestor followed by a gene loss in Ophiostomatales (red vertical bar and open circle). An additional gene transfer from Magnaporthales to *Colletotrichum* (red arrow) is sufficient to explain the phylogenetic conflict (Panel D). Possible gene presence in other Sordariomycetes species (e.g., Sordariales in Panel G) and therefore earlier gene origin (grey circles) does not

change this conclusion (Panel D). In a scenario of vertical inheritance and gene loss (Panel E), an instance of gene duplication is required in the Sordariomycetes common ancestor followed by 6 gene losses (red vertical bars) in paralog A leaving genes in only Diaporthales and 5 gene losses in paralog B leaving genes in only Magnaporthales and *Colletotrichum*. All together, 1 gene duplication and 11 gene losses are required in this second scenario (Panel E). For simplicity, we do not consider the additional gene duplication and gene losses (grey circles in Panel E) that may be required because of the gene presence in additional Sordariomycetes species such as Sordariales (e.g., *Podospora anserine* in Panel G). Sordariales are sister to the monophyletic group containing Magnaporthales, Ophiostomatales, and Diaporthales (Panel B). Taken together, the changes that are required to explain this category of gene tree (Panel A) are 1 gene transfer + 1 gene loss assuming HGT *versus* 1 gene duplication + 11 gene losses assuming vertical transmission and gene loss. The same principle applies to the gene trees, in which the Magnaporthales-*Colletotrichum* monophyletic group is sister to Hypocreales (Panel F). Hypocreales (e.g., *Fusarium*) are sister lineage to the *Colletotrichum* + *Verticillium* group (Panel B and Fig. 1B in main text).

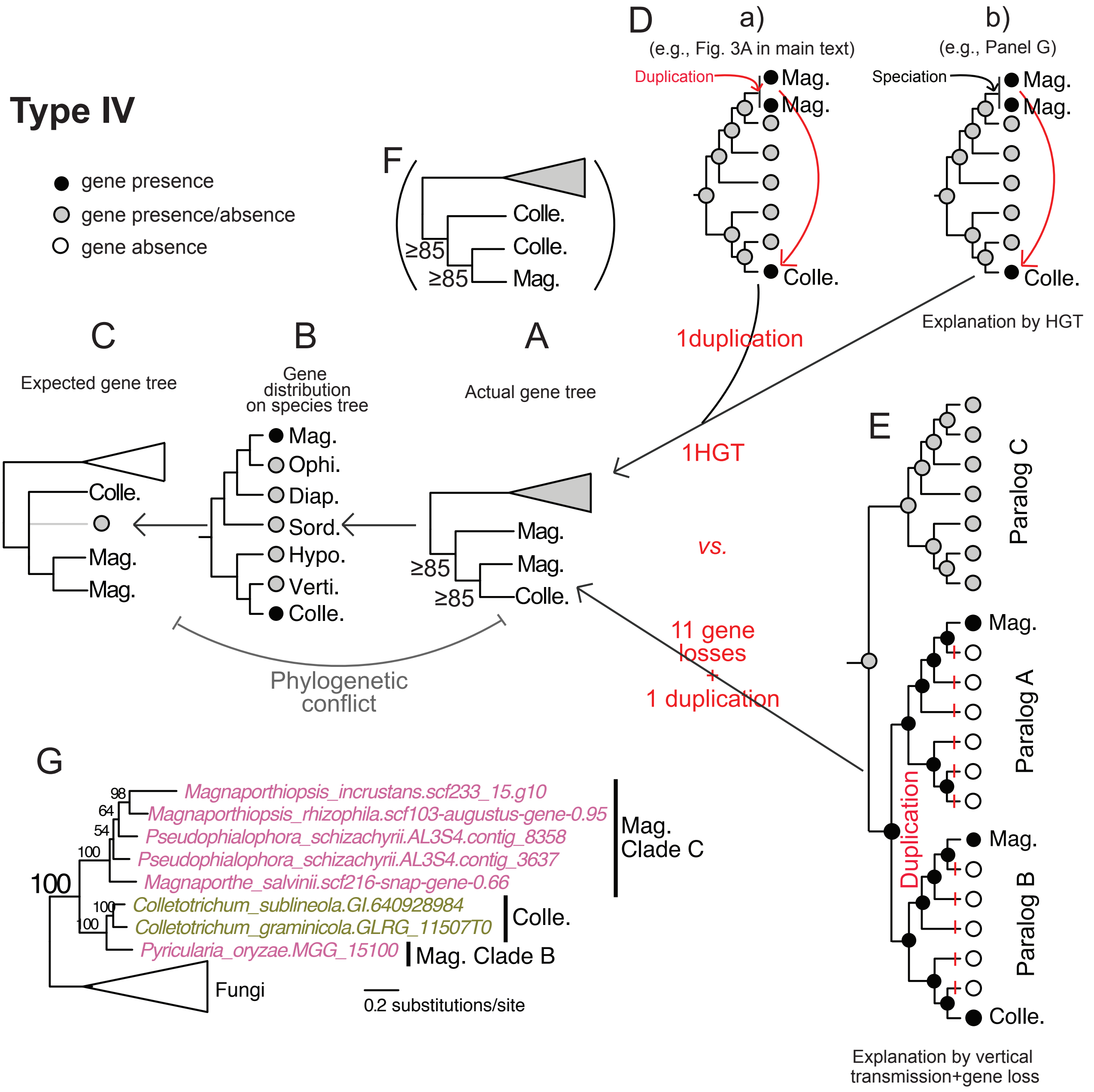

Fig. 4. Taxonomic mapping and phylogenetic conflicts in Type IV HGTs. In this category, Magnaporthales-*Colletotrichum* forms a sister group relationship to Magnaporthales (or *Colletotrichum* is nested among Magnaporthales, Panel A). The two nodes uniting Magnaporthales and *Colletotrichum* both have ≥85% UFboot support (Panel A). The remaining tree is collapsed and is presented using a grey triangle (Panel A). In the Sordariomycetes species tree, Magnaporthales correspond to a lineage that is separated from *Colletotrichum* by 5 different lineages (Panel B and Fig. 1B in main text). The black circles indicate the presence of the gene in Magnaporthales and *Colletotrichum*. Grey circles indicate that the gene may or may not be present in the corresponding lineage (Panel B). In either case, a generalized gene tree (Panel C) can be inferred given the gene distribution in the species tree (Panel B). Magnaporthales genes should form a monophyletic group (via speciation or lineage-specific gene duplication) with exclusion of *Colletotrichum* genes and other Sordariomycetes species (if any, the gray branch and circle). Consequently, a phylogenetic conflict arises between the actual gene tree (Panel A) and the inferred gene tree (Panel C). In a scenario allowing HGT (Panel D), Magnaporthales split

via either lineage-specific gene duplication (Scenario A in Panel D, see Fig. 3A in main text for an example) or speciation (Scenario B in Panel D, and Panel G). Magnaporthales phylogeny is well resolved in Ref. 16. This is followed by gene transfer from Magnaporthales to *Colletotrichum* (the red arrow). Thus, one instance of HGT (and probably one duplication) is sufficient to explain the phylogenetic conflict (Panel D). The possible earlier gene origin and gene presence in other Sordariomycetes species (indicate by grey circles) do not change the conclusion (Panel D). In a scenario with vertical inheritance and gene loss (Panel E), one gene duplication is required in the Sordariomycetes common ancestor followed by 6 gene losses (red vertical bars) in paralog A leaving only genes in Magnaporthales and 5 gene losses in paralog B leaving genes in only Magnaporthales and *Colletotrichum*. Taken together, 1 gene duplication and 11 gene losses are required in this second scenario (Panel E). For simplicity, we do not consider the additional gene duplication and gene losses that may be required in the case of the gene presence in other Sordariomycetes species (grey circles in Panel E). Taken together, the changes that are required to explaining this category of gene tree (Panel A) are 1 gene transfer + 1 (possible) gene duplication when assuming HGT *versus* 11 gene losses + 1 gene duplication when assuming vertical transmission and gene losses. The same principle applies to gene trees in which Magnaporthales-*Colletotrichum* monophyletic group are sister to *Colletotrichum* (or Magnaporthales are nested among *Colletotrichum*, Panel F).
