# Supplementary material for: Extensive horizontal gene transfers between plant pathogenic fungi
Source: BMC Biol. 2016 May 23;14:41. doi: 10.1186/s12915-016-0264-3 (PMC4876562; doi:10.1186/s12915-016-0264-3)
Supplement: Additional file 10: — Sordariomycete genome assemblies used for horizontal gene transfer validation. (PDF 102 kb) [file 12915_2016_264_MOESM10_ESM.pdf]

Additional file 10. Sordariomycetes genome assemblies used for the HGT validation procedure.

No.	Order	Species	GenBank assembly accession
1	Glomerellales	*Verticillium alfalfae*	GCA_000150825.1
2	Glomerellales	*Verticillium dahliae*	GCA_000952015.1
3	Glomerellales	*Verticillium longisporum*	GCA_001268165.1
4	Glomerellales	*Verticillium tricorpus*	GCA_000732205.1
5	Hypocreales	*Aciculosporium take*	GCA_000222935.2
6	Hypocreales	*Acremonium chrysogenum*	GCA_000769265.1
7	Hypocreales	*Atkinsonella hypoxylon*	GCA_000729835.1
8	Hypocreales	*Atkinsonella texensis*	GCA_001008105.1
9	Hypocreales	*Balansia obtecta*	GCA_000709145.1
10	Hypocreales	*Beauveria bassiana*	GCA_000770705.1
11	Hypocreales	*Beauveria rudraprayagi*	GCA_000733645.1
12	Hypocreales	*Claviceps fusiformis*	GCA_000223055.1
13	Hypocreales	*Claviceps paspali*	GCA_000223175.2
14	Hypocreales	*Claviceps purpurea*	GCA_000347355.1
15	Hypocreales	*Clonostachys rosea*	GCA_000963775.2
16	Hypocreales	*Cordyceps militaris*	GCA_000225605.1
17	Hypocreales	*Dactylonectria macrodidyma*	GCA_000935225.1
18	Hypocreales	*Epichloe amarillans*	GCA_000877375.1
19	Hypocreales	*Epichloe aotearoae*	GCA_000729855.1
20	Hypocreales	*Epichloe baconii*	GCA_000729845.1
21	Hypocreales	*Epichloe brachyelytri*	GCA_000222915.1
22	Hypocreales	*Epichloe bromicola*	GCA_001008065.1
23	Hypocreales	*Epichloe elymi*	GCA_000315335.1
24	Hypocreales	*Epichloe festucae*	GCA_000226195.1
25	Hypocreales	*Epichloe gansuensis*	GCA_000222895.2
26	Hypocreales	*Epichloe gansuensis*	GCA_000309355.1
27	Hypocreales	*Epichloe glyceriae*	GCA_000225285.2
28	Hypocreales	*Epichloe sp_AL9924*	GCA_000729825.1
29	Hypocreales	*Epichloe sylvatica*	GCA_001008265.1
30	Hypocreales	*Epichloe typhina*	GCA_000222955.2
31	Hypocreales	*Epichloe uncinata*	GCA_001043855.1
32	Hypocreales	*Escovopsis weberi*	GCA_001278495.1
33	Hypocreales	*Fusarium avenaceum*	GCA_000769215.1
34	Hypocreales	*Fusarium circinatum*	GCA_000876485.1
35	Hypocreales	*Fusarium fujikuroi*	GCA_001023045.1
36	Hypocreales	*Fusarium graminearum*	GCA_000966635.1
37	Hypocreales	*Fusarium langsethiae*	GCA_001292635.1
38	Hypocreales	*Fusarium nygamai*	GCA_001262555.1
39	Hypocreales	*Fusarium oxysporum*	GCA_000733055.2
40	Hypocreales	*Fusarium pseudograminearum*	GCA_000974265.1
41	Hypocreales	*Fusarium sp_JS626*	GCA_000966865.1
42	Hypocreales	*Fusarium temperatum*	GCA_001513835.1
43	Hypocreales	*Fusarium verticillioides*	GCA_000149555.1
44	Hypocreales	*Fusarium virguliforme*	GCA_000585705.1
45	Hypocreales	*Hirsutella minnesotensis*	GCA_000956045.1
46	Hypocreales	*Hirsutella thompsonii*	GCA_000472125.2
47	Hypocreales	*Hypocrella siamensis*	GCA_000731825.1
48	Hypocreales	*Isaria farinosa*	GCA_000733625.1
49	Hypocreales	*Metarhizium acridum*	GCA_000187405.1
50	Hypocreales	*Metarhizium album*	GCA_000804445.1
51	Hypocreales	*Metarhizium anisopliae*	GCA_000814975.1
52	Hypocreales	*Metarhizium brunneum*	GCA_000814965.1
53	Hypocreales	*Metarhizium guizhouense*	GCA_000814955.1
54	Hypocreales	*Metarhizium majus*	GCA_000814945.1
55	Hypocreales	*Metarhizium robertsii*	GCA_000187425.2
56	Hypocreales	*Nectria haematococca*	GCA_000151355.1
57	Hypocreales	*Neonectria ditissima*	GCA_001306435.1
58	Hypocreales	*Ophiocordyceps sinensis*	GCA_000448365.1
59	Hypocreales	*Ophiocordyceps unilateralis*	GCA_001272575.1

60	Hypocreales	*Paecilomyces hepiali*	GCA_001455915.1
61	Hypocreales	*Periglandula ipomoeae*	GCA_000222875.2
62	Hypocreales	*Pochonia chlamydosporia*	GCA_000411695.1
63	Hypocreales	*Purpureocillium lilacinum*	GCA_001468795.1
64	Hypocreales	*Stachybotrys chartarum*	GCA_001021365.1
65	Hypocreales	*Stachybotrys chlorohalonata*	GCA_000732775.1
66	Hypocreales	*Tolypocladium inflatum*	GCA_000421905.1
67	Hypocreales	*Tolypocladium ophioglossoides*	GCA_001189435.1
68	Hypocreales	*Tolypocladium sp_Salcha_MEA-2*	GCA_000750145.2
69	Hypocreales	*Tolypocladium sp_Sup5_PDA-1*	GCA_000750105.3
70	Hypocreales	*Torrubiella hemipterigena*	GCA_000825705.1
71	Hypocreales	*Trichoderma atroviride*	GCA_000963795.1
72	Hypocreales	*Trichoderma gamsii*	GCA_001481775.1
73	Hypocreales	*Trichoderma hamatum*	GCA_000331835.2
74	Hypocreales	*Trichoderma harzianum*	GCA_000733085.2
75	Hypocreales	*Trichoderma longibrachiatum*	GCA_000332775.1
76	Hypocreales	*Trichoderma parareesei*	GCA_001050175.1
77	Hypocreales	*Trichoderma reesei*	GCA_000513815.1
78	Hypocreales	*Trichoderma virens*	GCA_000170995.2
79	Hypocreales	*Ustilaginoidea virens*	GCA_000965225.1
80	Microascales	*Ceratocystis albifundus*	GCA_000813685.1
81	Microascales	*Ceratocystis fimbriata*	GCA_000389695.2
82	Microascales	*Ceratocystis manginecans*	GCA_000712455.1
83	Microascales	*Ceratocystis platani*	GCA_000978885.1
84	Microascales	*Huntiella moniliformis*	GCA_000712465.1
85	Microascales	*Huntiella omanensis*	GCA_000833645.1
86	Microascales	*Huntiella savannae*	GCA_001483325.1
87	Microascales	*Scedosporium apiospermum*	GCA_000732125.1
88	Microascales	*Scedosporium aurantiacum*	GCA_000812075.1
89	Microascales	*Thielaviopsis musarum*	GCA_001513885.1
90	Microascales	*Thielaviopsis punctulata*	GCA_000968615.1
91	Diaporthales	*Chrysoporthe austroafricana*	GCA_001051155.1
92	Diaporthales	*Chrysoporthe cubensis*	GCA_001282315.1
93	Diaporthales	*Diaporthe ampelina*	GCA_001006365.1
94	Diaporthales	*Diaporthe aspalathi*	GCA_001447215.1
95	Diaporthales	*Diaporthe longicolla*	GCA_000498855.2
96	Diaporthales	*Ophiognomonia clavigignenti*	GCA_000220195.1
97	Diaporthales	*Valsa mali*	GCA_000818155.1
98	Ophiostomatales	*Grosmannia clavigera*	GCA_000143125.1
99	Ophiostomatales	*Leptographium lundbergii*	GCA_001455505.1
100	Ophiostomatales	*Leptographium procerum*	GCA_000806385.1
101	Ophiostomatales	*Ophiostoma novo-ulmi*	GCA_000317715.1
102	Ophiostomatales	*Ophiostoma piceae*	GCA_000410735.1
103	Ophiostomatales	*Sporothrix brasiliensis*	GCA_000820605.1
104	Ophiostomatales	*Sporothrix pallida*	GCA_000710705.1
105	Ophiostomatales	*Sporothrix schenckii*	GCA_000961545.1
106	Sordariales	*Chaetomium globosum*	GCA_000143365.1
107	Sordariales	*Chaetomium thermophilum*	GCA_000221225.1
108	Sordariales	*Madurella mycetomatis*	GCA_001275765.1
109	Sordariales	*Myceliophthora thermophila*	GCA_000226095.1
110	Sordariales	*Neurospora africana*	GCA_000604205.2
111	Sordariales	*Neurospora crassa*	GCA_000786625.1
112	Sordariales	*Neurospora pannonica*	GCA_000604225.2
113	Sordariales	*Neurospora sublineolata*	GCA_000604185.2
114	Sordariales	*Neurospora terricola*	GCA_000604245.2
115	Sordariales	*Neurospora tetrasperma*	GCA_000213195.1
116	Sordariales	*Sordaria macrospora*	GCA_000182805.2
117	Togniniales	*Phaeoacremonium minimum*	GCA_000392275.1
118	Xylariales	*Daldinia eschscholtzii*	GCA_000261445.1
119	Xylariales	*Eutypa lata*	GCA_000349385.1
120	Xylariales	*Hypoxylon sp_E7406B*	GCA_000931505.1
121	Xylariales	*Pestalotiopsis fici*	GCA_000516985.1
122	Xylariales	*Rosellinia necatrix*	GCA_001445595.1
123	Xylariales	*Xylaria sp_JS573*	GCA_000966885.1
