# Supplementary material for: Extensive horizontal gene transfers between plant pathogenic fungi
Source: BMC Biol. 2016 May 23;14:41. doi: 10.1186/s12915-016-0264-3 (PMC4876562; doi:10.1186/s12915-016-0264-3)
Supplement: Additional file 12: — Vertebrate and fungal genome data underlying Fig. 1a. (PDF 239 kb) [file 12915_2016_264_MOESM12_ESM.pdf]

Additional file 12. Vertebrate and fungal genome data underlying Fig. 1A.

No.	Species	NCBI Genome ID Number
1	human *Homo sapiens*	51
2	opossum *Monodelphis domestica*	220
3	chicken *Gallus gallus*	111
4	frog *Xenopus tropicalis*	80
5	Spotted gar *Lepisosteus oculatus*	10597
6	elephant shark *Callorhinchus milii*	689
7	sea lamprey *Petromyzon marinus*	287
8	tunicate *Ciona intestinalis*	49
9	*Magnaporthiopsis incrustans*	* Ref. 16
10	*Nakataea oryzae*	* Ref. 16
11	*Pyricularia oryzae*	62
12	*Ophioceras dolichostomum*	* Ref. 16
13	*Pseudohelonectia lignicola*	* Ref. 16
14	*Grosmannia clavigera*	2302
15	*Neurospora crassa*	19
16	*Thielavia terrestris*	10696

* No available NCBI Genome ID Number.
